# A parthenogenetic quasi-program causes teratoma-like tumors during aging in wild-type *C. elegans*

**DOI:** 10.1038/s41514-018-0025-3

**Published:** 2018-06-13

**Authors:** Hongyuan Wang, Yuan Zhao, Marina Ezcurra, Alexandre Benedetto, Ann F. Gilliat, Josephine Hellberg, Ziyu Ren, Evgeniy R. Galimov, Trin Athigapanich, Johannes Girstmair, Maximilian J. Telford, Colin T. Dolphin, Zhizhou Zhang, David Gems

**Affiliations:** 1 School of Chemistry and Chemical Engineering, Harbin Institute of Technology, Harbin, 150001 China; 2 Institute of Healthy Ageing, University College London, London, UK; 3 School of Biological & Chemical Sciences, Queen Mary University of London, London, UK; 4 Division of Biochemical and Life Sciences, Faculty of Health and Medicine, Lancaster University, Lancaster, UK; 5 Department of Genetics, Evolution and Environment, University College London, London, UK; 6 Institute of Pharmaceutical Science, King’s College London, London, UK

## Abstract

A long-standing belief is that aging (senescence) is the result of stochastic damage accumulation. Alternatively, senescent pathology may also result from late-life, wild-type gene action (i.e., antagonistic pleiotropy, as argued by Williams) leading to non-adaptive run-on of developmental programs (or *quasi-programs*) (as suggested more recently by Blagosklonny). In this study, we use existing and new data to show how uterine tumors, a prominent form of senescent pathology in the nematode *Caenorhabditis elegans*, likely result from quasi-programs. Such tumors develop from unfertilized oocytes which enter the uterus and become hypertrophic and replete with endoreduplicated chromatin masses. Tumor formation begins with ovulation of unfertilized oocytes immediately after exhaustion of sperm stocks. We show that the timing of this transition between program and quasi-program (i.e., the onset of senescence), and the onset of tumor formation, depends upon the timing of sperm depletion. We identify homology between uterine tumors and mammalian ovarian teratomas, which both develop from oocytes that fail to mature after meiosis I. In teratomas, futile activation of developmental programs leads to the formation of differentiated structures within the tumor. We report that older uterine tumors express markers of later embryogenesis, consistent with teratoma-like activation of developmental programs. We also present evidence of coupling of distal gonad atrophy to oocyte hypertrophy. This study shows how the Williams Blagosklonny model can provide a mechanistic explanation of this component of *C. elegans* aging. It also suggests etiological similarity between teratoma and some forms of senescent pathology, insofar as both are caused by quasi-programs.

## Aging: Runaway programs are a cause of aging

The main cause of disease worldwide is aging, a process long suspected to result from damage accumulation. However, new work from a team led by David Gems at University College London describes a different type of cause: quasi-programs, or the futile run-on biological programs in later life, supporting a recently proposed, alternative theory about aging. The study focuses on the short-lived worm *C. elegans*, which during aging develop very large tumors in the uterus. The authors show how such tumors develop due to futile attempts by unfertilized eggs to develop by switching on programs of embryogenesis. In mammals, similar runaway programs cause teratomas, a type of benign tumor of grotesque appearance. This work provides new insights about the nature of aging as a disease process, suggesting that it is to an extent teratoma-like.

## Introduction

Although aging (i.e., senescence) is now the main cause of mortal disease worldwide, the causes of senescence (i.e., the etiologies of diseases of aging) remain poorly understood. Senescence has been much studied using simple, experimentally tractable model organisms such as the nematode *Caenorhabditis elegans*. Major advances have been made in terms of identifying genes and pathways that affect *C. elegans* lifespan, but less so in terms of understanding the proximate mechanisms of aging that such genes influence.^1,2^

The presence of genes where loss of function increases lifespan implies that wild-type gene action is a cause of aging. This is consistent with Williams’ evolutionary principle of antagonistic pleiotropy (AP): that natural selection can favor alleles that enhance fitness in early life even if they promote pathology later in life. This can occur because natural selection declines with age after the onset of reproduction.^3,4^

**Fig. 1:**
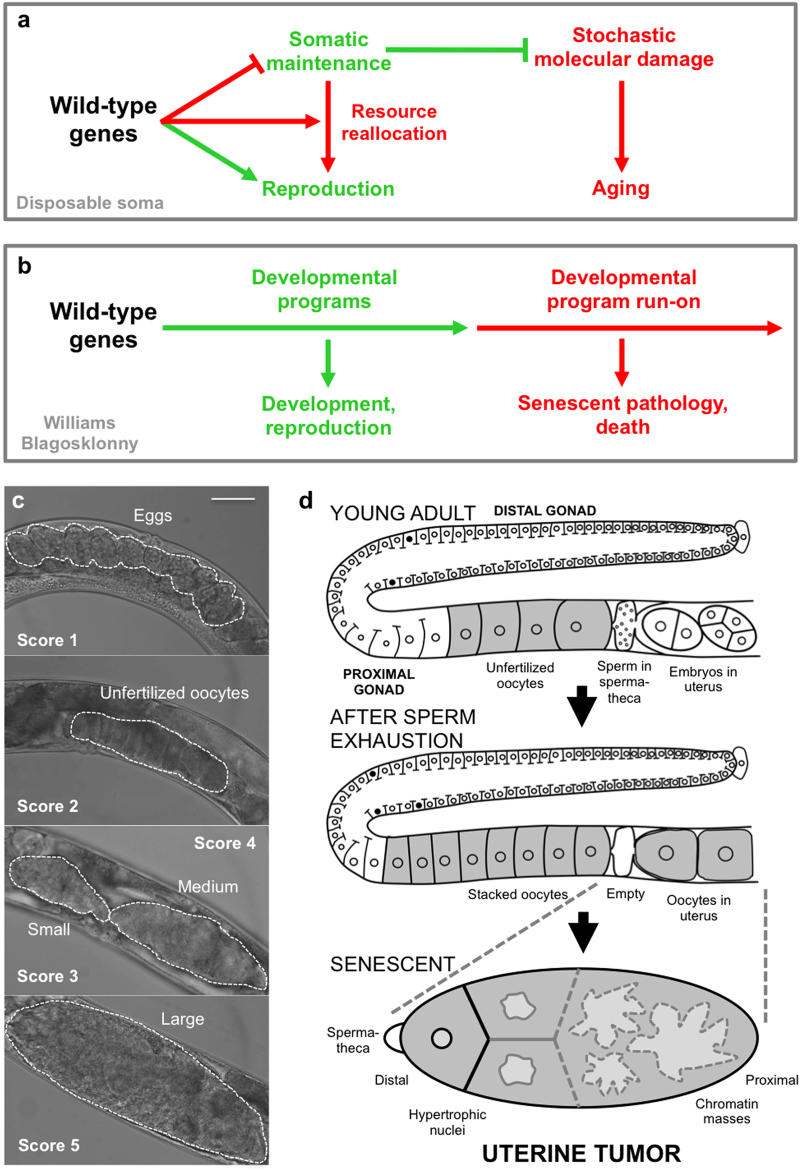
Theories of aging and uterine tumor development. **a**, **b** Alternative models for how wild-type genes cause senescence (pathogenic action of alleles exhibiting antagonistic pleiotropy). **a** Disposable soma model. Wild-type genes promote reproduction at the expense of somatic maintenance processes that prevent the damage that causes aging. **b** Williams Blagosklonny model. Continued action of wild-type genes in later life leads to run-on of developmental programs (quasi-programs) causing development of senescent pathology. **c**, **d** Senescent uterine tumors in *C. elegans* hermaphrodites. **c** Stages of development of uterine tumors. Dotted lines delineate uterine contents. Scale bar, 50 μm. **d** Schematic representation of uterine tumor development, consistent with Williams Blagosklonny model. As argued in this study, the point of origin of pathophysiology is immediately after fertilization with the last sperm in the spermatheca; this is the point of transition from program to quasi-program. Red, promoting senescent pathology

Critical to understanding aging is identification of the mechanisms by which AP is enacted, i.e., of the pathophysiology of AP, and there are several theories about this. The disposable soma theory suggests that late-life costs reflect insufficient resource investment into somatic maintenance mechanisms that reduce the molecular damage that causes aging (Fig. 1a).^5^ *C. elegans* aging studies regularly make reference to this theory.^6–11^

In an alternative model suggested more recently, the predominant cause of life-limiting senescent pathology is not molecular damage but, rather, non-adaptive continuation of developmental processes in later life (Fig. 1b).^12–14^ To describe such processes the term “quasi-programs” was proposed, to emphasize that although these are complex, orchestrated, developmental processes they are nonetheless non-adaptive. Here developmental is meant loosely, to include e.g., adult reproductive development and wound healing. To employ an analogy: “if you left water running after taking a bath, then a “program” for filling the bathtub would become a “quasi-program” to flood your apartment. Unlike a program, a quasi-program has no purpose (therefore, this does not contradict evolutionary theory)”.^13^ Mammalian examples of quasi-programmed processes (or quasi-programs) include the promotion of cancer in late life by the senescence-associated secretory phenotype (SASP),^15^ and of atherosclerotic plaque formation by inflammation, leading to cardiovascular disease.^16^ In each case functions that promote fitness in early life are activated in later life with pathogenic consequences.

Here we use this framework of ideas to investigate aging in *C. elegans*. Accordingly, we focus on understanding how wild-type gene action generates senescent pathology. This involves a shift in approach from what is more traditional in *C. elegans* biogerontology, in that we use senescent pathology rather than lifespan as a metric of aging: arguably, discovering the origins of senescent pathologies is key to understanding aging. Our approach involves a reasonable assumption: that when *C. elegans* die of old age, it is as the result of diseases of aging. Moreover, not all late-life disease promotes mortality, and the identity of life-limiting pathologies will vary between individuals, culture conditions and species. By this view, understanding the determinants of lifespan in *C. elegans* is less important in terms of applicability to human aging than understanding the causes of senescent pathology.

Compared to the genetics of lifespan, the biology of diseases of aging in *C. elegans* has been relatively neglected, apart from a few pioneering studies.^6,17–19^ Wild-type hermaphrodites exhibit a range of senescent pathologies whose severity and rapidity of development suggests the action of quasi-programs,^20^ and which involve atrophy (e.g., intestine, body wall muscles and gonad) and hypertrophy (e.g., uterus, some neurons and body cavity steatosis). The most striking hypertrophic pathology in aging hermaphrodites is the uterine tumors (also referred to as tumor-like masses or oocyte clusters).^11,21,22^ These arise from the germline of *C. elegans* hermaphrodites, which initially generates sperm that are stored in the spermatheca, but then switches to oocyte production. Oocytes are fertilized as they pass through the spermatheca until sperm stocks are depleted. After that, some unfertilized oocytes pass into the uterus where they undergo major cellular hypertrophy to form tumors so large that they can fill the entire body cavity in the *C. elegans* mid-body (Fig. 1c). Uterine tumors develop in all wild-type hermaphrodites but, despite their large size, they appear not to contribute to age-related mortality under standard culture conditions: preventing their development does not increase lifespan.^21,23^

*C. elegans* uterine tumors appear to result from quasi-programs. It was long ago observed that endomitosis is the “normal outcome” of entry of unfertilized oocytes into the uterus after sperm depletion.^24,25^ In such oocytes there occur multiple rounds of DNA replication and nuclear envelope breakdown but no karyokinesis or cytokinesis. This is because the mitotic centriole, normally supplied by sperm, is missing.^25^ Such cell cycle run-on leads to polyploidy, nuclear hypertrophy and, it has been postulated, eventually to uterine tumors^11,18,19^ (Fig. 1d).

The goal of this study is to better understand the pathophysiological origins of *C. elegans* uterine tumors, and to investigate whether they are caused by embryogenetic quasi-programs. This hypothesis drew on existing knowledge about uterine tumors from previous studies,^11,18,21,22^ in particular that by McGee et al.^19^, which examined the full course of tumor development. To this end, we have further characterized the developmental pathology of uterine tumors with an emphasis on morphometric analysis, and conducted tests to probe the role of several pathophysiological mechanisms.

## Results

### Correspondence between nuclear hypertrophy and uterine tumor size

In the previous study by McGee et al.^19^ tumors were studied in fixed animals stained with the DNA stain 4′,6-diamidino-2-phenylindole (DAPI) or with pararosaniline and methylene blue. To obtain further detail of the biology of tumor development, we used quantitative analysis of images obtained using Nomarski and epifluorescence microscopy. In the latter, fluorescent markers were used, since it is difficult to study the later stages of tumor development using light microscopy, due to their optical opacity and disorganized structure.

**Fig. 2:**
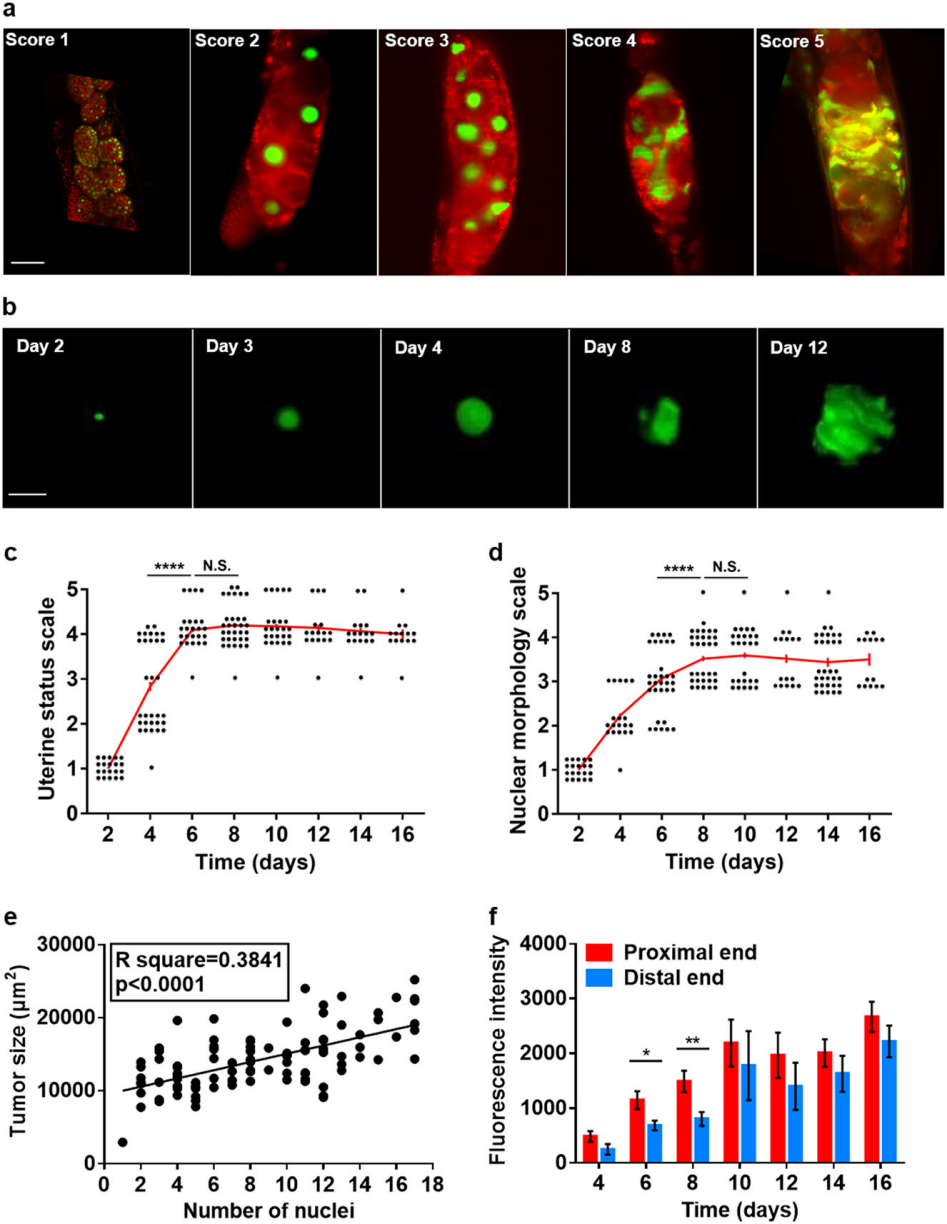
Further characterization of senescent uterine tumors. **a** Nuclear hypertrophy during tumor development (representative images of different animals). Scale bar, 25 μm. **b** Development of nuclear hypertrophy. Scale bar, 15 μm. **c** Uterine tumor development during aging. Uterine status scale: class 1, normal uterus containing eggs (day 1 adult). Class 2, slightly abnormal uterine contents, but no tumor visible. Class 3, small tumor. Class 4, medium sized tumor. Class 5, large tumor, filling body cavity and squashing the intestine. **d** Changes in nuclear morphology during tumor aging. Nuclear morphology scale (predominant morphology in tumor): class 1, normal sized, spherical nuclei. Class 2: moderately enlarged, spherical nuclei. Class 3: very large nuclei becoming non-spherical. Class 4: very large nuclei with frequent branching. Class 5: nuclei merged into large masses of chromatin. **c**, **d** day 0 is L4 stage; each point represents one tumor, and the same individuals were scored for each parameter; Wilcoxon–Mann Whitney test, ****p < 0.0001. **e** Linear regression analysis of number of oocyte nuclei and tumor size. **f** Nuclear hypertrophy is initially more marked in the proximal half of the tumor. Measured as HIS-58::GFP fluorescence intensity (mean ± s.e.m.); unpaired *t*-test, *p < 0.05; **p < 0.01. Sample sizes on each day: *n* = 9–24

For the purposes of morphometric characterization of tumor development, we used a strain with germline-expression of both HIS-58::GFP to mark chromatin, and mCherry::PH to mark cell membranes. DAPI staining confirmed that HIS-58::GFP fluorescence closely corresponds to nuclear DNA content and the presence of DNA masses (Figure S1a,b). Our aim was to characterize the development of nuclear hypertrophy, and its relationship to cellular hypertrophy, and the nature of the transition from orderly spherical nuclei to disordered DNA masses. Casual observation of images obtained using selective plane illumination microscopy (SPIM) confirmed the occurrence of marked nuclear hypertrophy with increasing age (Fig. 2a, b).

A semi-quantitative approach was used to characterize the dynamics of such hypertrophy, scoring nuclear status from 1 (normal sized, spherical nucleus) to 5 (nuclei merged into large masses of chromatin). The main period of tumor growth was between days 2 and 6 (Fig. 2c), as previously described.^21^ During the initial phase of nuclear growth (day 2–4), nuclear morphology remained normal (spherical), but from day 6 it became progressively irregular in appearance (Fig. 2b, d). With increasing hypertrophy nuclear masses developed protrusions and then large, branching extensions (Fig. 2b, d), which by day 8 became so large, extensive and random in structure that it became difficult to distinguish individual nuclei, which merged into amorphous masses of chromatin (Fig. 2a, b; see Video S1, S2 for 3D views of changes in nuclear morphology). Nuclear morphology and tumor size were positively correlated (Table S2), likely reflecting correspondence between nuclear size and morphological complexity. Although tumors reach a maximal size by day 6, chromatin masses continue to increase in size and morphological complexity until day 8 (Fig. 2c, d). A positive correlation was also seen between tumor size (day 6) and the number of oocytes within each tumor (as inferred from the number of oocyte nuclei) (Fig. 2e); thus, tumor size is partly determined by number of oocytes in the uterus, and partly by the extent of oocyte hypertrophy.

Nuclear hypertrophy was initially more marked at the proximal end of the tumor, nearest the vulva (Fig. 2f), consistent with the greater age and more advanced patho-developmental stage of proximal cells. This proximal-distal gradient in hypertrophy disappeared in fully developed tumors (Figure S1c) as all nuclei approached their upper limit of hypertrophy.

Previous quantitative analysis by PCR showed a ~4-fold increase in genomic DNA copy number that coincided approximately with the appearance of DAPI-stained masses and uterine tumors.^18,19^ To test this in more detail we compared the timing of quantified age increases in intra-uterine chromatin (using HIS-58::GFP) and total worm DNA content (using PCR) and observed an overall correspondence (Figure S1d,e). The initial decline in DNA content could reflect disappearance of embryos, and the late decline in GFP fluorescence could result from fluorophore breakdown. These results confirm that the late-life increase in DNA content is attributable to uterine tumor development, as previously proposed.^18,19^

### Similarities between *C. elegans* uterine tumors and mammalian ovarian teratomas

**Fig. 3:**
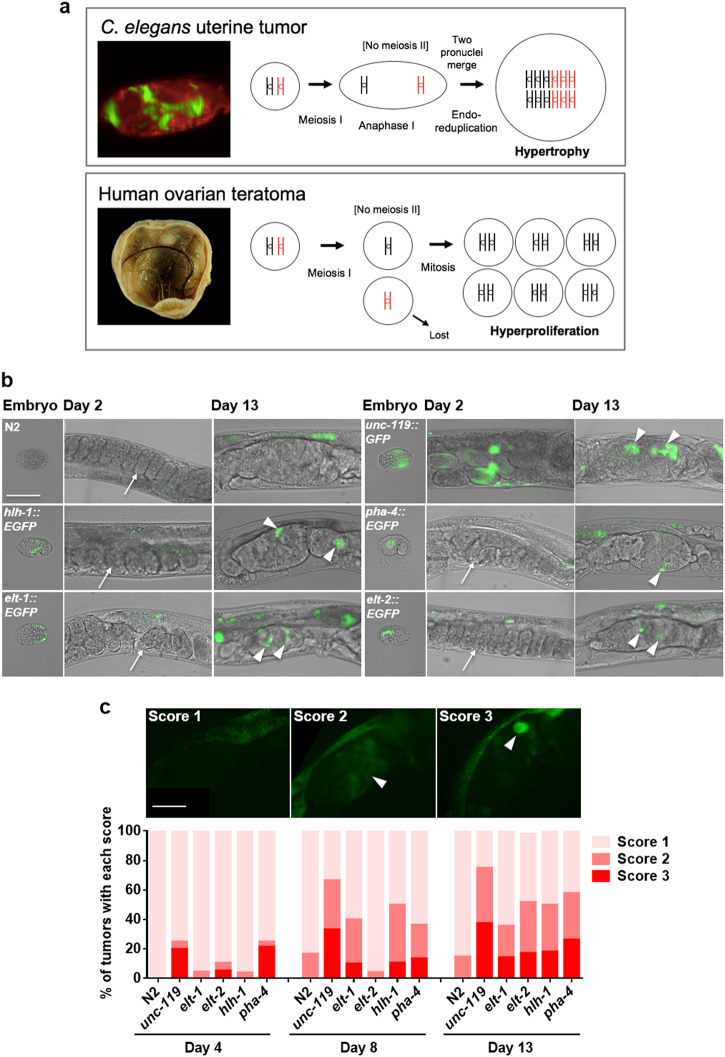
C. e*legans* uterine tumors are teratoma-like. **a** *C. elegans* uterine tumors and human ovarian teratomas are etiologically similar in that both originate from action in unfertilized immature oocytes of quasi-programs initiated after failure of meiosis II. **b**, **c** Expression of embryonic reporters in later stage uterine tumors. **b** Selected images of early and late stage tumors (epifluorescence microscopy). Note no fluorescence in early embryos (arrows). Expression was detected in older tumors (arrowheads). Scale bar, 50 μm. **c** Frequency of tumors expressing fluorescent markers at different levels (1–3 scale). Scale bar 50 μm. Score of reporter fluorescence within tumor: score 1, no fluorescence; score 2, weak fluorescence; score 3, strong fluorescence. GFP fluorescence in uterine tumors (arrowheads: fluorescence). Scorer was not blind to the treatment group. All trials, *n* ≥ 19. Teratoma image courtesy of E. Uthman http://web2.airmail.net/uthman/specimens/images/teratoma.html

Can one learn about human disease etiology by studying senescent pathology in *C. elegans*? This depends on the existence of shared pathophysiologies. Background reading led us to identify a mammalian pathology with an etiology resembling that of *C. elegans* uterine tumors: ovarian teratoma. In *C. elegans* the final stages of oocyte maturation, including completion of meiosis I and II, occur after fertilization, but in oocytes that remain unfertilized meiosis ceases after anaphase I.^26^ Thus, *C. elegans* uterine tumors develop from diploid immature oocytes (Fig. 3a). Similarly, mammalian ovarian teratomas arise from initiation of embryonic programs in unfertilized oocytes. Here maturing oocytes complete meiosis I but do not undergo meiosis II, and are thus diploid but homozygous at most loci.^27,28^

Teratomas are a form of benign tumor generated by activation of developmental programs in the wrong context. In humans, 10–20% of abnormal ovarian neoplasms are teratomas. Within such tumors growth and differentiation can give rise to multiple tissue types and complex structures, most commonly skin, hair, muscle, and cartilage^29^ (Fig. 3a). Other forms of teratoma can form more complex structures including bone, teeth and even partial fetuses; the grotesque appearance of some teratomas accounts for their name (*tera*, Greek: monster).

In mammalian ovarian teratomas inappropriate expression of embryogenetic programs leads to pathological embryonic differentiation and morphogenesis;^27^ thus teratomas may be understood to result from quasi-programs. We wondered whether anything similar occurs within *C. elegans* uterine tumors. To this end, we first examined expression of five genes expressed in various embryonic and adult tissues but not in oocytes, *elt-1* (sperm-producing germ line)*, elt-2* (intestinal expression)*, hlh-1* (muscle)*, pha-4* (pharyngeal and intestinal expression), and *unc-119* (pan-neuronal), using GFP or EGFP reporters, in tumors on days 2, 4, 6, 8, and 13 of adulthood. A previous study saw no muscle or neuronal reporter expression in wild-type, polyploid unfertilized oocytes from 2 to 2.5 day old adults.^30^ Consistent with this, we saw no reporter gene expression in the early stages of tumor development. However, in late-stage tumors, after chromatin mass formation, expression of all reporters was seen, *unc-119* and *pha-4* from day 4 and *elt-1, elt-2* and *hlh-1* from day 8 or 13 (Fig. 3b, c). Expression was variable, occurring in some tumors but not others, and varying in intensity in different regions of expression-positive tumors.

Analysis of reporter expression in tumors was complicated by the fact that uterine tumors sometimes contain fertilized eggs embedded within them. In some cases, such embedded embryos appear to be developing normally, but others are degenerated (Figure S1f). Such embedded embryos were rarely seen in tumors of older animals. Examination of fluorescent regions within tumors using bright field microscopy did not reveal the presence of embedded embryos (data not shown), confirming that tissue derived from unfertilized oocytes was expressing embryonic genes.

The earlier appearance of *punc-119::GFP* expression is notable since embryonic expression of this reporter occurs earlier than the others, and was visible in embryos even before egg laying (Fig. 3b). This suggests that the order of expression of embryonic markers may follow the same sequence as that in embryogenesis, consistent with recapitulation of embryonic programs within the tumor. To test this further we combined *punc-119::mCherry* and *pelt-2::EGFP* reporters in the same strains, and confirmed the earlier expression of *punc-119::mCherry* (Figure S2a,b). Notably, the different markers were expressed at different locations within tumors (Figure S2b). Red fluorescence from *punc-119::mCherry* was distinguishable from naturally occurring red autofluorescence (see below), as the former was far brighter and more widely distributed (Figure S2c).

In mutationally-induced *C. elegans* germline teratomas, as in mammalian teratomas, germ cells transdifferentiate into various somatic tissues including intestine, muscle and neurons.^30^ It seemed to us unlikely that actual tissue differentiation occurs in wild-type uterine tumors, since these are formed from a small number of large, hypertrophic cells. To explore this we looked for the presence of several markers of tissue differentiation: blue autofluorescent gut granules (intestine), expression of *unc-119::GFP* in filamentous processes (neuron), and expression of *myo-3::GFP* in a striated pattern (muscle), but in no case was differentiation detected (data not shown). MYO-3::GFP fluorescence was sometimes seen in embedded embryos (Figure S2d). To verify that such fluorescence issued from embedded embryos rather than teratomas, we examined a fertilization defective *rrf-3(b26); edIs6 [unc-119::GFP+rol-6(su1006)]* strain. *rrf-3(b26)* largely abrogated MYO-3::GFP fluorescence within tumors (Figure S2e).

Overall, these results imply that older uterine tumors to some extent recapitulate embryonic gene expression programs, but that this does not lead to full blown teratomas with embryonic differentiation, perhaps due to the absence of cell proliferation. Thus, *C. elegans* uterine tumors resemble mammalian ovarian teratomas in that (a) both originate from diploid cells resulting from a failure in meiosis II in oogenesis (Fig. 3a) and (b) both develop due to inappropriate switching on of embryogenetic programs (i.e., from parthenogenetic quasi-programs).

### Evidence that tumor development is initiated by sperm depletion

To understand a disease one wants, ideally, to identify its initial cause. For example, the original cause of malaria is the bite of an *Anopheles* mosquito that transmits protozoa of the genus *Plasmodium*. Long-standing questions in research on aging are: what is the origin of senescence? When and how exactly does it begin? Does such a starting point even exist? In the case of *C. elegans* uterine tumors this question may be answered: the likely point of origin of pathology, equivalent to the mosquito’s bite, is the departure of the last functional sperm from the spermatheca. This marks is the point of transition from program to quasi-program. Shortly thereafter, the first unfertilized oocyte enters the uterus and begins to undergo endomitosis, and the pathogenetic process begins that eventually generates gross, teratoma-like senescent pathology.

According to this account it should be possible to accelerate or delay the development of uterine tumors by bringing forward or delaying sperm depletion, i.e., altering the timing of the mosquito bite (the transition from program to quasi-program). To test this we first examined *fog-2(q71)* mutants which, due to germline feminization, do not produce sperm thereby changing hermaphrodites into females.^31^ Tumors were found to develop earlier in *fog-2* females than in N2 hermaphrodites (Figure S3a; day 1–3 of adulthood). By contrast, tumor formation was suppressed by mating in both *fog-2* females and N2 hermaphrodites (Figure S3b-d). Thus, altering the timing of the transition from program to quasi-program alters the rate of development of the senescent pathology that the quasi-program generates. Tumors also failed to grow in *fem-3(q20)* mutants which produce sperm but not oocytes (Figure S3e), confirming that tumors develop from oocytes.

### Identification of genes contributing to tumor development

If uterine tumors result from embryogenetic quasi-programs, then this predicts that many genetic determinants of key functions in early embryogenesis (e.g., DNA replication, the cell cycle and protein synthesis) (Table S1) will promote tumor development. To explore this we used RNAi to inhibit function of candidate genes, testing for suppression of tumor growth. To increase sensitivity in these assays, we measured tumor size (cross-sectional area) rather than scoring pathology level.

**Fig. 4:**
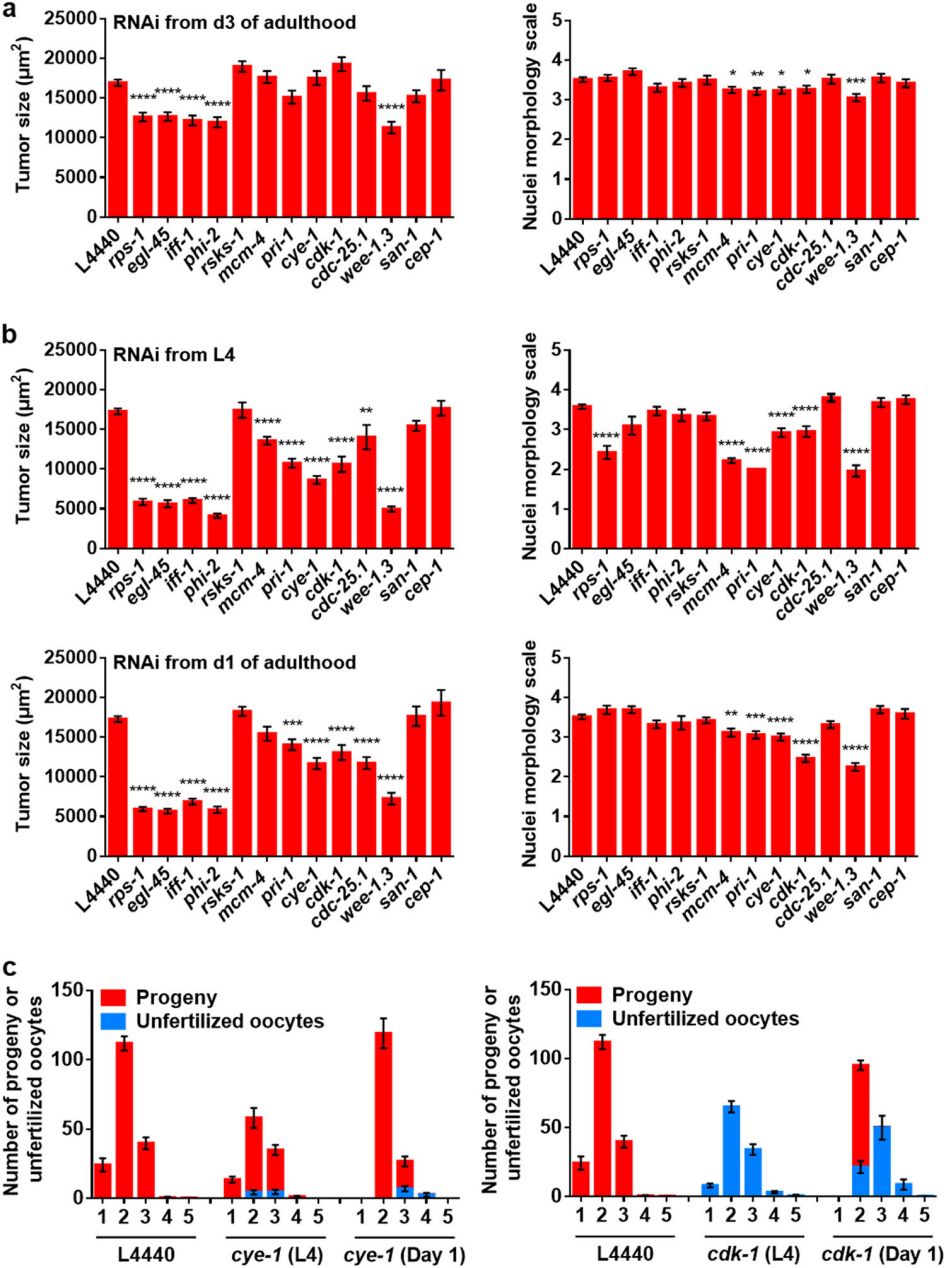
Knockdown of genes required for early embryogenesis reduces tumor growth. **a**, **b** Effects of whole worm RNAi initiated at different ages on nuclear hypertrophy and tumor size (mean ± s.e.m.), measured on day 8 of adulthood. Dunnett multiple comparison test, Wilcoxon–Mann Whitney test, *p < 0.05; **p < 0.01; ***p < 0.001; ****p < 0.0001. All trials, *n* ≥ 17. **a** RNAi initiated from day 3. **b** RNAi initiated from L4 and day 1. **c** Effects of *cye-1* and *cdk-1* RNAi on fertility schedule (progeny and unfertilized oocytes). All trials, *n* ≥ 9. Data are mean ± s.e.m

We first performed whole worm RNAi. Interventions that retard oocyte production rate are predicted to delay sperm depletion, and consequently the onset of tumor development. We therefore first tested RNAi initiated on day 3 of adulthood, to try to limit the effect of gene knockdown to the period after sperm depletion. Four genes involved in protein synthesis (*rps-1*, *egl-45*, *iff-1*, and *phi-2*) also served as positive controls, since RNAi was known to inhibit germline development.^32^ Here RNAi reduced tumor size, showing efficacy of RNAi within the uterus, but did not affect nuclear size (Fig. 4a). Of other knockdowns, only that of *wee-1.3* reduced tumor size. However, in most cases a significant reduction of nuclear hypertrophy was seen.

The lack of effects on tumor size could reflect weak reduction of gene function. We therefore tested RNAi initiated earlier, on L4 and day 1 of adulthood, and this resulted in major reductions in both tumor size and nuclear hypertrophy in most cases (Fig. 4b). This included inhibition of genes involved in DNA replication (e.g., *mcm-4* and *pri-1*) and cell cycle promoters (e.g., *cye-1* and *cdk-1*). RNAi did not reduce body size, even when initiated at L4 (Figure S4a), nor was tumor size correlated with body size in wild type (Figure S4b), thus RNAi effects on tumors were not attributable to altered worm size. Again RNAi of most protein synthesis genes caused reduction of tumor growth but not of nuclear hypertrophy (apart from *rps-1* RNAi, which reduced both).

To check that reduction in tumor size were not merely the result of a delay in sperm depletion, we examined the effect of RNAi of selected genes on reproduction (Fig. 4c). RNAi of *cye-1* and *cdk-1* from L4 and day 1 did not delay the reproductive schedule, indicating that the timing of sperm depletion was unaffected.

We then verified that RNAi effects on tumor growth reflect gene action in the germline by using *ppw-1(pk1425)* which causes the germline to be RNAi resistant, and *rrf-1(ok589)* which attenuates somatic but not germline RNAi (Figure S4c,d). *ppw-1* blocked RNAi effects on tumor growth for genes encoding DNA replication and cell cycle function but, notably, tumor growth was still reduced by RNAi of several genes promoting protein synthesis, *egl-45*, *iff-1*, and *rps-1* (Figure S4c). One possibility is that this is due to reduced intestinal synthesis of yolk which, as described below, feeds tumor growth. By contrast, *rrf-1(ok589)* had little effect on RNAi inhibition of tumor growth (Figure S4d).

These results provide evidence that the function of wild-type genes specifying functions required for early embryonic development, including the cell cycle and protein synthesis, promote growth and reproduction in early life and senescent uterine tumor development after reproduction, another example of antagonistic pleiotropy as run-on.^3,14^ This is clearest with respect to nuclear hypertrophy; with respect to overall tumor size, the possibility that RNAi reduces the number of oocytes entering the uterus has not been ruled out.

### Uterine tumor hypertrophy is coupled to distal gonadal atrophy

**Fig. 5:**
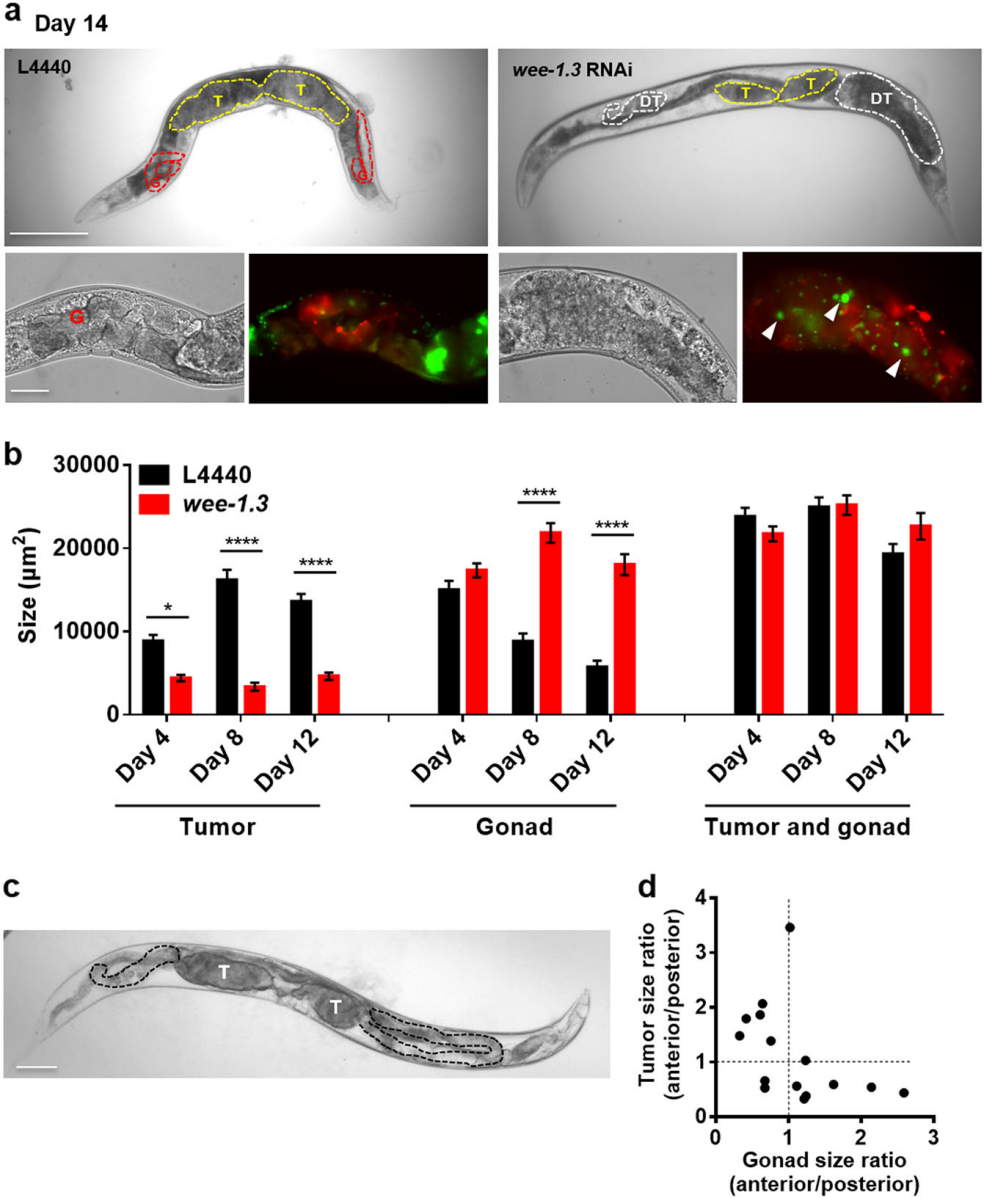
Evidence for coupling of uterine tumor growth and distal gonad atrophy. **a** Distal gonad tumors resulting from *wee-1.3* RNAi (CDK inhibitory kinase). Yellow dotted line delineates uterine tumors, white dotted lines distal gonad hypertrophic tumors, and red dotted lines gonad. Bottom right: The size and number of HIS-58::GFP-labeled nuclei indicate that these tumors, like uterine tumors, result from cellular hypertrophy rather than hyperplasia. Arrowhead, hypertrophic nucleus. T uterine tumor, DT distal gonad hypertrophic tumor. Scale bar, 50 μm. **b** Quantitation of effects of *wee-1.3* RNAi on uterine and distal hypertrophic tumor size; note lack of change in combined size. Sidak multiple comparison test, *p < 0.05; ****p < 0.0001. Sample sizes: L4440, day 4, *n* = 12, day 8, *n* = 13, day 12, *n* = 20; *wee-1.3* RNAi, day 4, *n* = 26, day 8, *n* = 14, day 12, *n* = 22. Data are mean ± s.e.m. **c**, **d** Asymmetry in gonad disintegration in animals with asymmetrical uterine tumor pairs. **c** Day 19 adult with asymmetric tumors (T), and asymmetric gonadal atrophy (delineated with black dotted line). Scale bar, 100 μm. T tumor. **d** Ratio of anterior tumor/posterior tumor size and gonad size within the same worm with asymmetric tumors (10 μM FUDR) on day 9 and day 10. *n* = 15

*wee-1.3* encodes a CDK inhibitory kinase, and we therefore expected that *wee-1.3* RNAi would enhance uterine tumor growth but, surprisingly, the opposite effect was seen (Fig. 4a, b). However, *wee-1.3* RNAi also resulted in a novel phenotype: development in the distal gonad of additional tumors formed, like uterine tumors, from a small number of large, hypertrophic cells with enlarged nuclei (Fig. 5a). This suggests that loss of CDK inhibition in the distal gonad leads to cellular hypertrophy.

One possibility is that development of distal gonad hypertrophic tumors somehow causes the observed reduction in uterine tumor size. Consistent with this, the sum of the mean cross-sectional area of uterine tumors and distal gonad was constant in day 4, day 8, and day 12 adults (Fig. 5b). However, in a comparison of the size of the two tumor types in individual worms, we were unable to detect a negative correlation (data not shown).

Next we asked whether anything similar might occur in wild-type hermaphrodites. In wild-type hermaphrodites, one uterine tumors develops at each distal end of the uterus. Anterior and posterior tumors are, on average, similar in size (Figure S4e), and tumors in a given worm are usually similar in size (Figure S4f). However, marked tumor size asymmetry occurs in a small fraction of worms (<5%). Interestingly, the majority (~80%) of those animals also show corresponding asymmetry in the degree of distal gonad atrophy (Fig. 5c, d). This suggests the presence of a common etiology promoting both uterine tumor growth and distal gonad atrophy; one possibility is that resources released by distal gonad atrophy support tumor growth, another that signals from the tumor promote atrophy of the attached distal gonad. Coupling of uterine tumor hypertrophy and distal gonad atrophy could explain how RNAi of *cdk-1* and *wee-1.3* both inhibit tumor growth despite their opposing effects on the cell cycle: by suppressing distal gonad atrophy, *wee-1.3* RNAi also suppresses uterine tumor growth.

### Quasi-programmed yolk uptake also contributes to tumor growth

**Fig. 6:**
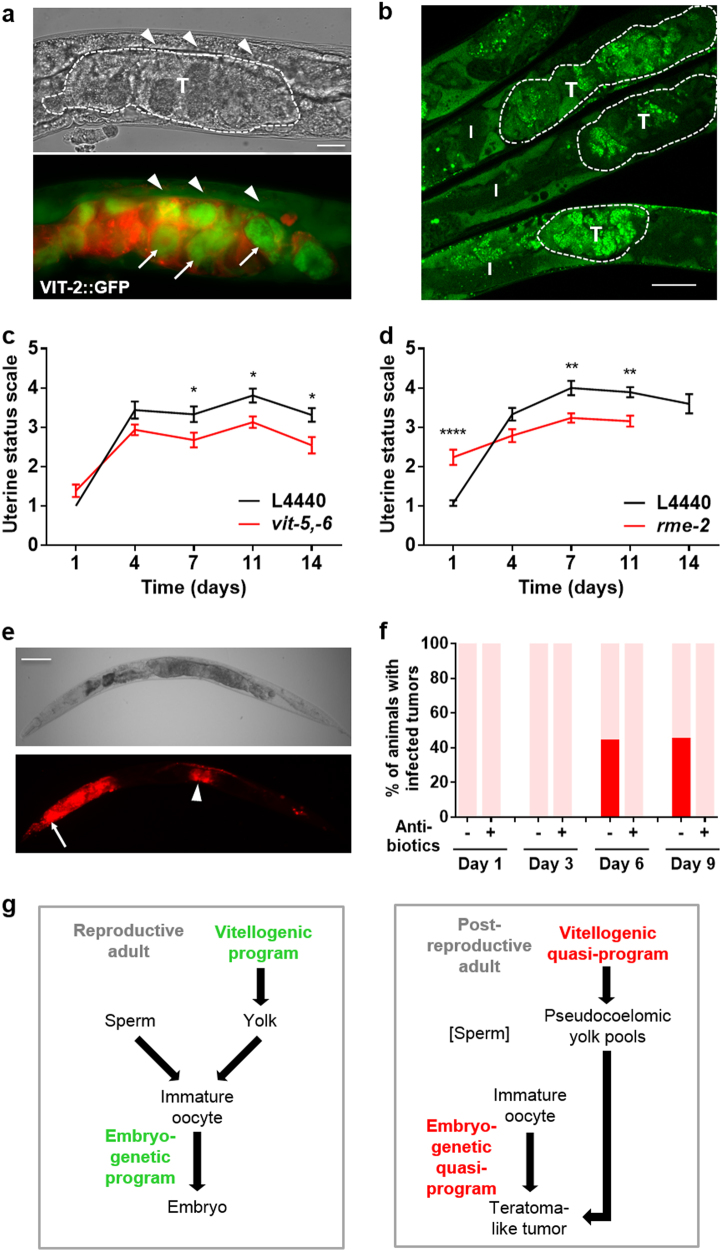
Additional uterine tumor etiologies: yolk uptake and bacterial infection. **a** VIT-2::GFP accumulation in uterine tumors (day 10 adult), with cell membranes marked with mCherry::PH. VIT-2::GFP accumulation in oocytes within tumors (arrows) and in yolky pools (arrowheads). T, uterine tumor. Scale bar, 25 μm. **b** Neutral lipid staining shows lipid accumulation within uterine tumors, delineated by dotted lines (day 8 adult, 15 μM FUDR). I intestine, T uterine tumor. Scale bar, 25 μm. **c** *vit-5,-6* RNAi reduces tumor size (mean ± s.e.m.). Summed data from 3 trials; Sidak multiple comparison test, *p < 0.05. Sample sizes on each day: *n* = 19–32. **d** *rme-2* RNAi reduces tumor size (mean ± s.e.m.). Summed data from 2 trials. **p < 0.01, ****p < 0.0001. Sample sizes on each day: *n* = 14–21 (*rme-2* RNAi, day 14, *n* = 5). **c**, **d** RNAi initiated at hatching. **e**, **f** Uterine tumors can develop bacterial infections. **e** *E. coli* expressing dsRed within uterine tumors (day 10 adult, necropsy). Arrow, infected pharynx. Arrowhead, infected tumor. Scale bar, 100 μm. **f** Bar graph showing frequency of worms with infected tumors, without or with carbenicillin (Fisher Scientific, 4800-94-6). Pink, uninfected; red, infected. **g** Scheme summarizing how programs that act to promote reproduction in young adults (left) become quasi-programs to promote pathology in older adults, after sperm depletion (right). Arrows indicate a contributory effect

Prior to ovulation, maturing oocytes import yolk (vitellogenin and yolk lipid) from the body cavity using the LDL receptor-like protein RME-2.^33,34^ Previous analysis of stained, fixed sections of aged hermaphrodites showed high levels of yolk between tumor cells in the uterus.^19^ This raises the possibility that futile yolk import by oocytes in the uterus contributes to tumor growth, e.g., by increasing their bulk through its presence, or by providing nutrients to support new biosynthesis within tumor cells. Consistent with this, tumors in a *C. elegans* strain expressing a GFP-tagged vitellogenin, VIT-2::GFP, show strong green fluorescence (Fig. 6a, Figure S5a,b; see Video S3 for 3D view). Moreover, staining of neutral lipids in fixed worms with the fluorescent dye Bodipy showed high levels of lipid within tumors (Fig. 6b).

To test this further, we used combined RNAi of *vit-5* and *vit-6* initiated at L4, which largely abrogates yolk accumulation in adults,^35^ and found that it reduced tumor size (Fig. 6c). RNAi of the *rme-2* yolk receptor from L4 had a similar effect (Fig. 6d). To test whether yolk uptake after ovulation contributes to tumor growth we compared effects of *vit-5,-6* RNAi initiated at L4, day 1, 3, or 6 of adulthood, and saw significant reduction in tumor size in all cases (Figure S6a, Table S3). We also detected an age increase in VIT-2::GFP fluorescence within tumors (Figure S6b). Increasing vitellogenin levels using the *bIs1* VIT-2::GFP transgene array did not increase tumor size (data not shown), perhaps because wild-type yolk levels are not limiting for tumor growth. Taken together these results support the view that yolky lipid pools that accumulate in the body cavity^6,17^ promote further pathology by feeding uterine tumors.

### Uterine tumors can develop bacterial infections

We also noted that wild-type *C. elegans* maintained on *E. coli* expressing the red fluorescent protein dsRed sometimes develop strong red fluorescence within tumors (Fig. 6e). This suggests that uterine tumors can become infected by the *E. coli* upon which *C. elegans* are routinely cultured. Examination of dsRed positive tumors using light microscopy confirmed the presence of *E. coli* within the tumors (data not shown). One possibility is that such infections are nourished by the rich yolky milieu of the tumor (Figure S6c). Such infections are prevented by treatment with the antibiotic carbenicillin (Fig. 6f), revealing another mechanism by which antibiotics reduce senescent pathology and, possibly, extend lifespan.^17^ Taken together, these findings illustrate how distinct etiologies (embryogenetic and vitellogenic quasi-programs, and bacterial infection) act together in the genesis of a major senescent pathology.

### Is the uterus a latent tumor niche?

Previous histological analysis implied that despite their large size, tumors remain largely within the uterus.^19^ To verify this quantitatively in live animals, we used a COG-1::GFP reporter that is expressed in the spermathecal-uterine valve, which marks the distal end of the uterus (and also the vulva). Tumors of 28 worms were examined on day 8 of adulthood and in all cases where spermathecal GFP was detectable, this marked the distal end of the tumor (Figure S7a,b).

That extra-uterine oocytes do not develop into tumors raises the possibility that the uterus possesses tumor niche properties that are latent prior to sperm depletion. The latent tumor niche is a disease etiology paradigm where an unintended juxtaposition of two tissues leads to induction of the development of pathology.^36^ This disease mechanism is thought to contribute to early stages of cancer, prior to mutation, and perhaps to senescent pathology,^36^ and is another form of quasi-program etiology. For example, if *C. elegans* mitotic germ cells are mislocated near the somatic gonadal sheath, the latter acts as a tumor niche due to Notch ligand secretion (including DSL-5), resulting in hyperplastic proximal tumors.^36^ In tests of Notch ligand gene knock-down and mutation, we observed that *dsl-5(ok588)* slows tumor growth without delaying the timing of sperm depletion (Figure S7c,d). However, the site of expression of *dsl-5* has proved difficult to determine^36^ (Supplemental Results). Moreover, reduction of Notch receptor function after sperm depletion using *glp-1(e2141ts)* did not reduce tumor size (Figure S7e) (for further details, see Supplemental Results). The possibility that a uterine tumor niche promotes oocyte hypertrophy warrants further investigation.

## Discussion

A central claim of the Williams Blagosklonny model (Fig. 1a) is that developmental quasi-programs promoted by late-life action of wild-type genes are a major cause of late-life pathology (i.e., of aging). Previous studies suggest a contributory role for quasi-programs to senescent pathologies in *C. elegans*. For example run-on of physiological apoptosis contributes to atrophy of the hermaphrodite gonad,^37^ and run-on of yolk production leads to yolk steatosis and, through gut-to-yolk biomass conversion, to intestinal atrophy.^6,35^ Here, we show how *C. elegans* senescent uterine tumors exemplify generation of senescent pathology by quasi-programs. Importantly, the main etiology defined here, a parthenogenetic quasi-program, is similar to that of human ovarian teratomas, demonstrating the conservation of quasi-programmed etiologies between *C. elegans* and humans. Thus, etiologies of native diseases of aging in *C. elegans* are relevant to mammalian disease.

### Uterine tumors illustrate how antagonistic pleiotropy can be enacted through quasi-programs

Mutation of the *daf-2* insulin/IGF-1 receptor suppresses uterine tumor development.^19^ Here *daf-2* exhibits antagonistic pleiotropy (AP), promoting development first of germline^38^ and then of tumors. Williams originally envisaged AP acting on numerous genes with discrete phenotypic effects (Figure S8a). From this perspective, the initial discovery of genes like *daf-2* that appear to affect the entire aging process was unexpected. This paradox was partially resolved with the realization that *daf-2* affects expression of numerous other genes, including many involved in somatic maintenance, consistent with the disposable soma theory.^39^ However, a better fit with uterine tumor pathobiology is Blagosklonny’s concept of quasi-program: a complex, developmental program that runs on, resulting in the development of pathology^13^ (Figure S8b). Here, plausibly, pathology results not passively from damage accumulation or failure of homeostasis, but rather actively from gene action (hyper-function; Fig. 1b). However, it remains possible that stochastic damage and disposable soma-type AP also contribute to uterine tumor etiology.^19^ For example, it was recently shown that absence of sperm increases protein aggregation in unfertilized oocytes,^40^ which might contribute to tumor pathophysiology.

### Cell cycle run-on causes uterine tumor development

Uterine tumor development appears to originate with run-on of endomitosis. This leads to polyploidy, nuclear hypertrophy and cellular hypertrophy. Increased ploidy promotes growth in many contexts, e.g., in the *C. elegans* hypodermis,^41^ and we postulate that by similar mechanisms increased oocyte ploidy drives cellular hypertrophy.

In mammals, cancer is considered to be primarily the consequence of somatic mutations affecting the control of cell proliferation. However, the age-increase in cancer rate is to some degree the result of the effect on aging on the cellular microenvironment.^15^ Moreover, some forms of cancer can originate with run-on rather than mutation, for example benign prostatic hyperplasia (BPH) in humans, which typically precedes prostate cancer, is the result of long-term testosterone exposure rather than mutation.^42^ Thus, the initial cause of BPH, like *C. elegans* uterine tumors, appears to be quasi-programs rather than damage.

### Similarities between uterine tumors and mammalian teratomas

*C. elegans* uterine tumors are pathophysiologically similar to mammalian ovarian teratomas in several respects. First, both result from a failure of meiosis II leading to formation of abnormal cells that are diploid derivatives of immature oocytes. Secondly, in both cases these cells make a botched attempt to form an embryo, through futile expression of embryogenetic developmental programs. In the case of *C. elegans* this gives rise to amorphous masses derived from hypertrophic, polyploid oocyte-derived cells. They show expression of genes associated with later embryonic development, but no detectable morphological differentiation, and no cell proliferation, in contrast to mammalian teratomas. A further similarity is that human ovarian teratomas are usually benign, and *C. elegans* uterine tumors do not increase late-life mortality.^21^

A difference between *C. elegans* uterine tumors and mammalian ovarian teratomas is that the former is a senescent pathology but not the latter, which instead occur largely during reproductive years (in humans between ~20–40 years of age).^43^ Nonetheless, the fact of the etiological features that they share—in broad terms, quasi-programs resulting from wild-type gene function—yields a grim insight into the nature of aging: that senescent pathology and teratoma are to some extent pathophysiologically equivalent. That is to say, insofar as senescent pathology is caused by quasi-programs, aging and teratoma are the same sort of disease; likewise, cholera and tuberculosis are the same sort of disease, insofar as both are caused by bacterial infection.

It was previously shown that disruption of P granules (germ granules) also caused de-repression of somatic gene expression in the germline, including *unc-119*.^44^ An unexplored possibility is that loss of P granules might trigger such de-repression in older tumors.

### Multiple etiologies of tumorigenesis

Uterine tumors illustrate how different etiologies combine to cause senescent pathology (summarized in Fig. 6g). Here the initial etiology is run-on of embryogenetic quasi-programs. Subsequently, a second pathology, yolk steatosis, contributes to tumor growth, in a distant parallel to the increased cancer risk caused by obesity in mammals. Thirdly, tumors can become infected with bacteria, and here again there are resemblances to mammalian pathogenesis, as follows.

The relationship between bacteria and cancer progression in humans is a long-standing topic of investigation. In some instances, bacteria appear to initiate or drive malignant alterations. For example, colorectal cancer is often associated with an abundance of *Streptococcus gallolyticus* and *Fusobacterium sp*, which are thought to trigger and drive cancer progression by causing persistent inflammation.^45,46^ Here, the anaerobic conditions and available nutrients within tumors provide an optimum environment for the growth of certain bacteria.^45^ However, bacterial infection of tumors is not necessarily detrimental to the host. Post-operative infection in lung cancer can actually improve patient survival,^47^ perhaps because bacteria compete for nutrients, secrete toxic proteins or help to initiate immune responses against tumor cells. Given the susceptibility of *C. elegans* uterine tumors to bacterial invasion as demonstrated here, they could serve as a model to study tumor–bacteria interactions.

## Conclusions

The main purpose of studying aging in *C. elegans* is to understand senescence, particularly in terms of its initial causes. This is part of the broader, general goal of biomedical research: to understand the etiologies of disease in order to be able to prevent and treat them.^48^ This study describes the primary mechanistic cause of one component of *C. elegans* aging, uterine tumor development. This exemplifies a class of diseases of aging caused by quasi-programs that appear to play a major role in the *C. elegans* aging process. We speculate that in *C. elegans*, quasi-programs are the predominant cause of senescence, along with other contributory causes, such as mechanical senescence and bacterial infection (this study).^49,50^ However, in mammalian aging molecular damage, particularly DNA damage, may play a greater role than in *C. elegans*.

## Materials and methods

### *C. elegans* culture and strains

Worms were cultured at 20 °C unless otherwise stated. Nematode strains used in this paper include N2 (wild-type) CGCH,^51^ CB4108 *fog-2(q71) V*, GA1932 *unc-119(ed.3) III; ltIs44 [pie-1p-mCherry::PH(PLC1deltal)+unc-119(+)]; ruIs32 [pie-1::GFP::H2B+unc-119(+)] III* and PS3662 *syIs63[cog-1::GFP+unc-119(+)]*. For full listing see Supplemental Experimental Procedures.

### Selective plane illumination microscopy (SPIM)

SPIM was used to make 3D reconstructions of *C. elegans* uterine tumors. Anaesthetized worms were transferred into a plastic capillary tube with 1.5% low melting point agarose, 0.03% levamisole and 0.5 μM FluoSphere microspheres (beads) (1:1000, F8813 from Life Technologies). FluoSphere beads allowed registration and 3D construction of 2D images taken from 5 angles. OpenSPIM was performed to take images as previously described.^52^

### Pathology measurements

Severity of uterine tumors were scored using a five stage classification as described.^21^ To measure different levels of nuclear hypertrophy we created another five stage classification. Score 1 denotes small, spherical early stage oocyte nuclei. Score 2 denotes larger but still spherical nuclei. Score 3 denotes larger nuclei with irregular morphology. Score 4 denotes highly hypertrophic nuclei with grossly abnormal phenotype (e.g., with major protrusions), such that some individual nuclei are barely distinguished. Score 5 denotes large chromatin masses where most individual nuclei can no longer be distinguished. Trials were initiated with L4 stage larvae (day 0).

### Statistical analysis

Statistical tests with appropriate assumptions on data distribution were used. Correlation analysis was performed using linear regression analysis. Non-parametric Wilcoxon–Mann Whitney test was performed to compare uterine status and nuclear morphology. Fluorescence intensity was measured using Volocity and Fiji software. Multiple comparison *t*-test was used to compare fluorescence intensity and tumor size.

For additional detail on methodology, see Supplemental Experimental Procedures.

### Data availability statement

All relevant data are available from the authors on request.

## Electronic supplementary material

Wang supporting information

Video S1

Video S2

Video S3

## Electronic supplementary material

**Supplementary information** accompanies the paper on the *npj Aging and Mechanisms of Disease* website (10.1038/s41514-018-0025-3).
