# Supplementary material for: A parthenogenetic quasi-program causes teratoma-like tumors during aging in wild-type C. elegans
Source: NPJ Aging Mech Dis. 2018 Jun 13;4:6. doi: 10.1038/s41514-018-0025-3 (PMC5998035; doi:10.1038/s41514-018-0025-3)
Supplement: Supplementary file 1 — Wang supporting information [file 41514_2018_25_MOESM1_ESM.pdf]

SUPPORTING INFORMATION

A parthenogenetic quasi-program causes teratoma-like tumors during aging in wild-type *C. elegans*

Hongyuan Wang, Yuan Zhao, Marina Ezcurra, Alexandre Benedetto, Ann F. Gilliat, Josephine Hellberg, Ziyu Ren, Evgeniy R. Galimov, Trin Athigapanich, Johannes Girstmair, Maximilian J. Telford, Colin T. Dolphin, Zhizhou Zhang and David Gems

Supporting Information listing

Figure S1 Quantitation of chromatin mass development in senescent uterine tumors.
Figure S2 Expression of markers of development in older uterine tumors.
Figure S3 Timing of sperm depletion affects timing of tumorigenesis.
Figure S4 Effect of RNAi on tumor size.
Figure S5 VIT-2::GFP accumulation in uterine tumors.
Figure S6 Relationship between other pathologies and uterine tumors.
Figure S7 Is the uterus a latent tumor niche?
Figure S8 Older vs newer ideas about the proximate action of antagonistic pleiotropy.
Figure S9 Red autofluorescence in senescent uterine tumors.

Table S1 Description of genes for RNAi experiment.
Table S2 Correlation between tumor size and nuclear morphology.
Table S3 Statistical analysis of effects of *vit-5,-6* RNAi on tumor size.

Video S1 3D view of nuclei within uterine tumors (light sheet microscopy [SPIM]).
Video S2 3D view of individual nuclei within tumors (light sheet microscopy [SPIM]).
Video S3 3D view of VIT-2::GFP accumulation in uterine tumor (confocal microscopy).

SUPPLEMENTAL RESULTS

lin-53 is not a suppressor of neuronal gene expression in uterine tumors

The status of germline chromatin prevents expression of somatic genes, and loss of the LIN-53 histone chaperone can promote neuronal differentiation within germline[1]. To probe the role of *lin-53* as a teratoma suppressor in uterine tumors, we tested effects of *lin-53* RNAi on tumor expression of an embryonic neuronal marker (*punc-119::GFP*). However, no effect of *lin-53* RNAi was detected (Figure S2f).

No correlation between pharyngeal and uterine infection

Under standard culture conditions some 40% of wild-type N2 hermaphrodites die as the result of pharyngeal infection with *E. coli* [2]. One possibility is that such worms have weaker organism-wide immunity which also leads to uterine tumor infection. To test this we examined worms on days 10 and 14, when major pharyngeal infections are relatively frequent, to see whether those with infected pharynxes had a higher frequency of infected tumors, but they did not (Figure S6d). Thus, independent mechanisms lead to bacterial infection of the pharynx and uterus.

Evidence of possible latent tumor niche properties in the uterus

That extra-uterine oocytes do not develop into tumors raises the possibility that the uterus might possess tumor niche properties that become manifest only after sperm depletion, when unfertilized, immature oocytes start to enter the uterus[3]. Proliferation of mitotic germ cells is normally promoted by Notch signaling from the distal tip cell[4]. However, mutations such as *pro-1(na48)* cause abnormal juxtaposition of mitotic germ cells with the gonad sheath, which stimulates mitosis and consequently the development of proximal tumors (the Pro phenotype)[5]. This has been attributed to production of Notch ligands by the gonad sheath, particularly APX-1, ARG-1 and DSL-5, since RNAi or mutation of these genes suppresses *pro-1(na48)* Pro, and *apx-1* and *arg-1* are expressed in the gonad sheath[3]. To explore this, we tested effects of *apx-1(or3), arg-1(ok3127)* and *dsl-5(ok588)* deletion mutations on uterine tumors and found that only *dsl-5(ok588)* reduced tumor development rate (Figure S7c; data not shown).

One possibility is that *dsl-5(ok588)* delays tumor development by reducing oocyte production rate and/or delaying sperm depletion. To test this we examined *dsl-5* mutant reproductive parameters, but detected no delay in the reproductive schedule or, after sperm depletion, reduction of unfertilized oocytes laid (Figure S7d), or level of stacking of oocytes in the proximal gonad[6] (data not shown). Thus, the delay in tumor growth in *dsl-5* mutants appears to be a consequence of slower tumor growth rather than delayed onset of tumor development.

A further possibility is that the effect of *dsl-5* on tumor growth is due to an overall delay in the aging process. To probe this we examined the effect of *dsl-5* on other senescent pathologies: pharyngeal deterioration, distal gonad atrophy, intestinal atrophy and yolk accumulation, as previously described[7,8]. The only effect detected was a slight increase in yolk accumulation (data not shown), possibly attributable to reduced yolk uptake by tumors.

APX-1 and ARG-1 are predicted to be membrane tethered, but DSL-5 is predicted to be secreted[9]. This suggests the possibility that DSL-5 is secreted into the uterine lumen. However, the site(s) of *dsl-5* expression remain unidentified: in a prior analysis of 10 lines with *pdsl-5::GFP* reporters including ~3.5 kb upstream of *dsl-5* no expression was detected[3]. This could mean that distant regulatory elements control *dsl-5* expression. Notably, *dsl-5* is the most downstream of six

closely spaced genes transcribed in the same orientation, the first two of which are part of a predicted operon. This suggests the possibility that *dsl-5* is downstream in a large operon, in which case thus the *dsl-5* promoter would lie far upstream of its open reading frame (ORF). To try to identify the site of *dsl-5* expression we used a counter-selection recombineering protocol[10] to replace the *dsl-5* ORF with GFP in the fosmid WRM0639dC09, which contains the entire six gene cluster. However, although multiple *C. elegans* transformants were generated containing the recombinant fosmid (4 germline, 5 transient, confirmed by PCR), no GFP was detected (data not shown). A remaining possibility is that *dsl-5* expression requires sequence elements that are downstream of the gene's translational start site, e.g. in intronic sequences.

Next we tested the effect on tumor growth of adult-specific abrogation of expression of the germline-expressed Notch receptor GLP-1, which also plays a major role in embryogenesis[11]. For this we used the temperature sensitive *glp-1(e2141)* mutant, which is virtually wild-type at 15°C[12]. *glp-1* hermaphrodites were shifted to the non-permissive temperature at several time points, including after self-sperm depletion (L4, day 2, 4, 6, 8), and tested for reduction in final tumor size (day 10), in comparison to similarly temperature-shifted N2 controls. Reduced tumor size was seen only upon upshift prior to sperm depletion (L4, day 2) (Figure S7e). This suggests that synthesis of GLP-1 in intra-uterine oocytes after sperm depletion does not promote tumor growth; however, it does not rule out a role for GLP-1 synthesized prior to the temperature shift. The possibility that a uterine tumor niche promotes oocyte hypertrophy warrants further investigation.

Age increase in red autofluorescence is attributable to uterine tumor growth

During epifluorescence analysis we noted the presence of red autofluorescence within the tumors of older wild-type hermaphrodites that was not present in earlier stage tumors (Figure S9a,b). Such red autofluorescence occurred in small patches at variable positions within the tumor. Notably, red autofluorescence is a biomarker of senescence in *C. elegans*[13]. This biomarker appears to be largely attributable to tumor patho-development since overall red fluorescence increased with age more strongly in *rrf-3(b26)* worms (tumors present) than *glp-4(bn2)* worms (tumors absent) (Figure S9c), and mainly occurred in the mid-body (Figure S9d).

SUPPLEMENTAL EXPERIMENTAL PROCEDURES

C. elegans culture and strains

Worms were cultured at 20˚C unless otherwise stated using standard methods as described previously[14]. Nematodes were cultured on *E. coli* OP50 or, in the case of RNAi trials, *E. coli* HT115. 5-fluoro-deoxyuridine (FUDR) was generally not used, except where specified. Nematode strains used in this paper include N2 (wild-type) CGCH [15], CB4037 *glp-1(e2141ts) III*, CB4108 *fog-2(q71) V* [16], DH26 *rrf-3(b26) II*, DP132 *edIs6 [unc-119::GFP + rol-6(su1006)] IV*, EU91 *apx-1(or3) V/nT1 [unc-?(n754) let-?](IV;V)*, GA1500 *bIs1[vit-2::GFP + rol-6(su1006)]*, GA1932 *unc-119(ed3) III; ltIs44 [pie-1p-mCherry::PH(PLC1deltal) + unc-119(+)]; ruIs32 [pie-1::GFP::H2B + unc-119(+)] III* [17], JK816 *fem-3(q20) IV* [18], NL3511 *ppw-1(pk1425) I,* OP37 *unc-119(ed3) III; wgIs37 [pha-4:: TY1::EGFP::3xFLAG + unc-119(+)]*, OP56 *unc-119(ed3) III; gaIs290 [elt-2::TY1::EGFP:: 3xFLAG(92C12) + unc-119(+)]*, OP64 *unc-119(ed3) III; wgIs64 [hlh-1::TY1::EGFP::3xFLAG + unc-119(+)]*, OP354 *unc-119(tm4063) III; wgIs354 [elt-1::TY1::EGFP::3xFLAG + unc-119(+)]*, PS3662 *syIs63 [cog-1::GFP + unc-119(+)]* [19], RW10006 *ruIs32* [*ppie-1::histone H2B::GFP*] *zuIs178* [*phis-72::his-72::GFP + unc-119(+)*] *unc-119(ed3) III* [20], MS1180 *irIs83 [pMM824 (unc-119::mCherry) + pMM768 (end-3(+)]*, RB798 *rrf-1(ok589) I* [21], RB797 *dsl-5(ok588) IV*, RB2304 *arg-1(ok3127) X*, and RW1596 *stEx30 [myo-3p::GFP::myo-3 + rol-6(su1006)].*

Construction of *dsl-5* transgenic lines

To construct *dsl-5* reporter lines including including potential distant gene-regulatory elements, we used the fosmid WRM0639dC09, which includes a cluster of six predicted genes, of which *dsl-5* is the most downstream. The open reading frame of the *dsl-5* gene was substituted with that of GFP using a counter selection recombineering protocol, as previously described[10]. Transgenic lines were generated by either microinjection or biolistic transformation, using *rol-6(su1006)* as a co-transformational marker. The presence of the fosmid in Rol transformants was confirmed by PCR.

Mating protocol

N2 or *fog-2(q71)* males were used for mating. A brief mating protocol was used to reduce the life-shortening effects of mating[22]. Sexes were separated at L4 stage and left overnight to develop into adults before being combined for mating. Animals were incubated at a ratio of 3:1 males to hermaphrodites/females for 5 hours (84 males + 28 hermaphrodites/females per plate), after which males were removed.

Progeny and unfertilized oocytes production assays

L4 larvae were cultured at a population density of one animal per plate, and transferred daily to fresh plates during the first five days of adulthood. After removal of the parent worm, each plate was then maintained at 20˚C for 1 day, and then larvae and unfertilized oocytes on the plate were counted using a dissecting microscope.

Microscopy

Nematodes were placed on 2% agarose pads and anesthetized with 0.2% levamisole. For most trials, Nomarski and epifluorescence microscopy was performed on a Zeiss Axioskop 2 Plus microscope connected to a Hamamatsu C10600 - Orca ER digital camera. Images were acquired and quantified using Volocity 6.3 software. 100x or 400x images are shown in this report. For viewing red

autofluorescence, a rhodamine filter was used (λ_{ex} 545/25 nm, λ_{em} 605/70 nm).

Selective plane illumination microscopy (SPIM) was used to make 3D reconstructions of *C. elegans* uterine tumors. Anaesthetized worms were transferred into a plastic capillary tube with 1.5% low melting point agarose, 0.03% levamisole and 0.5 μm sized FluoSphere microspheres (beads) (1:1000, F8813 from Life Technologies). Agarose embedding and anesthetic kept the animals immobile during imaging, and FluoSphere beads allowed registration and 3D construction of 2D images taken from 5 angles. We used a 1 ml BD Plastikpak (REF 300013) syringe to mount the plastic capillaries into the OpenSPIM chamber full of M9 buffer. OpenSPIM was performed to take images as previously described[23]. Acquired data was processed using Fiji software. The beads registration algorithm and the multi-view deconvolution plugin were performed for reconstruction of 3D structure[24,25].

DAPI staining and quantitation of genomic copies

Nuclei of uterine tumors were stained with the DNA-binding dye 4', 6'-diamidino-2-phenylindole (DAPI). Worms of different ages were fixed with methanol and incubated on ice, then washed with M9 buffer and stained with 500 ng/μl DAPI staining in darkness for 30 min. Finally, worms were washed again with M9 buffer before imaging. Genomic copy number was estimated using real-time PCR as described[26].

Bodipy staining

Lipid staining was performed in fixed worms as described[27], except that animals were manipulated in 15μL droplets within parafilm micro-wells. Worms were washed 2x by transferring them successively into two droplets of M9, and then transferred to a drop of 2% paraformaldehyde solution for 15-20min and frozen/thawed 3x at -80°C/room temperature (RT). They were then carefully washed 3x by transferring them into fresh M9 droplets. Worms were then transferred to 1μg/mL BODIPY 493/503 (Invitrogen) in M9 for 1-2hr at RT in darkness. Lastly, they were washed 3x in M9 droplets and mounted for imaging (λ_{ex} 488 nm, λ_{em} 505-575 nm) using a Zeiss LSM710 confocal microscope.

Pathology scoring system

Severity of uterine tumors was scored using a five stage classification as previously described[28]. Score 1 denotes a uterus containing fertilized eggs or oocytes of normal size and morphology. Score 2 denotes a uterus containing unfertilized oocytes only, with a slightly abnormal appearance. Score 3 and score 4 denote a small tumor and a large tumor, respectively. Score 5 denotes a very large tumor which fills the body cavity in mid-body region.

To measure different levels of nuclear hypertrophy we created another five stage classification. Score 1 denotes small, spherical early stage oocyte nuclei. Score 2 denotes larger but still spherical nuclei. Score 3 denotes larger nuclei with irregular morphology. Score 4 denotes highly hypertrophic nuclei with grossly abnormal phenotype (e.g. with major protrusions), such that that some individual nuclei are barely distinguished. Score 5 denotes large chromatin masses where most individual nuclei can no longer be distinguished.

To describe expression of embryonic reporters at different levels we used a three stage classification system. Score 1 denotes no reporter fluorescence in the tumor. Score 2 denotes weak reporter fluorescence in the tumor. Score 3 denotes strong reporter fluorescence in the tumor.

RNA-mediated interference

The *E. coli* feeding strains used for RNAi were obtained from the Ahringer RNAi strain library. DNA sequencing was used to verify the identity of all plasmid inserts. RNAi treatment was performed as described previously[29]. Worms were raised on *E. coli* HT115 and transferred to plates seeded with RNAi feeding strains at the L4 stage, or on day 1, 3 or 6 of adulthood. *E. coli* HT115 containing the empty plasmid vector L4440 was used as a control.

Statistical analysis

Correlation analysis was performed using linear regression analysis. The non-parametric Wilcoxon-Mann Whitney test was performed to compare uterine status and nuclear morphology. Fluorescence intensity was measured using Volocity and Fiji software. A multiple comparisons t test was used to compare fluorescence intensity and tumor size.

Supplemental References

1 Tursun, B., Patel, T., Kratsios, P. & Hobert, O. Direct conversion of c. elegans germ cells into specific neuron types *Science* **311**, 304-308 (2011).

2 Zhao, Y. *et al*. Two forms of death in aging Caenorhabditis elegans. *Nat. Commun.* **8**, 15458 (2017).

3 McGovern, M., Voutev, R., Maciejowski, J., Corsi, A. K. & Hubbard, E. J. A "latent niche" mechanism for tumor initiation. *Proc. Natl Acad. Sci. USA* **106**, 11617-11622 (2009).

4 Henderson, S., Gao, D., Lambie, E. & Kimble, J. lag-2 may encode a signaling ligand for the GLP-1 and LIN-12 receptors of C. elegans. *Development* **120**, 2913-2924 (1994).

5 Killian, D. J. & Hubbard, E. J. Caenorhabditis elegans germline patterning requires coordinated development of the somatic gonadal sheath and the germ line. *Dev. Biol.* **279**, 322-335 (2005).

6 Jud, M., Razelun, J., Bickel, J., Czerwinski, M. & Schisa, J. A. Conservation of large foci formation in arrested oocytes of Caenorhabditis nematodes. *Dev. Genes. Evol.* **217**, 221-226 (2007).

7 de la Guardia, Y. *et al*. Run-on of germline apoptosis promotes gonad senescence in C. elegans. *Oncotarget* **7**, 39082-39096 (2016).

8 Ezcurra, M. *et al*. Autophagy-dependent gut-to-yolk biomass conversion generates visceral polymorbidity in aging C. elegans. *bioRxiv*, doi.org/10.1101/234419 (2017).

9 Chen, N. & Greenwald, I. The lateral signal for LIN-12/Notch in C. elegans vulval development comprises redundant secreted and transmembrane DSL proteins. *Dev. Cell* **6**, 183-192 (2004).

10 Hirani, N. *et al*. A simplified counter-selection recombineering protocol for creating fluorescent protein reporter constructs directly from C. elegans fosmid genomic clones. *BMC Biotechnology* **13**, 1 (2013).

11 Priess, J. in *WormBook* (ed The C. elegans Research Community) (2005).

12 Kodoyianni, V., Maine, E. M. & Kimble, J. Molecular basis of loss-of-function mutations in the glp-1 gene of Caenorhabitis elegans. *Mol. Biol. Cell* **3**, 1199-1213 (1992).

13 Pincus, Z., Mazer, T. C. & Slack, F. J. Autofluorescence as a measure of senescence in C. elegans: Look to red, not blue or green. *Aging* **8**, 889-898 (2016).

14 Brenner, S. The genetics of *Caenorhabditis elegans*. *Genetics* **77**, 71-94 (1974).

15 Gems, D. & Riddle, D. L. Defining wild-type life span in *Caenorhabditis elegans*. *J. Gerontol. A Biol. Sci. Med. Sci.* **55**, B215-B219 (2000).

16 Schedl, T. & Kimble, J. *fog-2*, a germ-line-specific sex determination gene required for hermaphrodite spermatogenesis in *Caenorhabditis elegans*. *Genetics* **119**, 43-61 (1988).
17 Kachur, T. M., Audhya, A. & Pilgrim, D. B. UNC-45 is required for NMY-2 contractile function in early embryonic polarity establishment and germline cellularization in C. elegans. *Dev. Biol.* **314**, 287-299 (2008).
18 Barton, M., Schedl, T. & Kimble, J. Gain-of-function mutations of fem-3, a sex-determination gene in Caenorhabditis elegans. *Genetics* **115**, 107-119 (1987).
19 Palmer, R. E., Inoue, T., Sherwood, D. R., Jiang, L. I. & Sternberg, P. W. Caenorhabditis elegans cog-1 locus encodes GTX/Nkx6.1 homeodomain proteins and regulates multiple aspects of reproductive system development. *Dev. Biol.* **252**, 202–213 (2002).
20 Praitis, V., Casey, E., Collar, D. & Austin, J. Creation of low-copy integrated transgenic lines in *Caenorhabditis elegans*. *Genetics* **157**, 1217-1226 (2001).
21 Kumsta, C. & Hansen, M. C. elegans rrf-1 mutations maintain RNAi efficiency in the soma in addition to the germline. *PLoS One* **7**, e35428 (2012).
22 Gems, D. & Riddle, D. L. Longevity in *Caenorhabditis elegans* reduced by mating but not gamete production. *Nature* **379**, 723-725 (1996).
23 Girstmair, J. *et al.* Light-sheet microscopy for everyone? Experience of building an OpenSPIM to study flatworm development. *BMC Dev. Biol.* **16**, 22 (2016).
24 Preibisch, S., Saalfeld, S., Schindelin, J. & Tomancak, P. Software for bead-based registration of selective plane illumination microscopy data. *Nat. Methods* **7**, 418-419 (2010).
25 Preibisch, S. *et al.* Efficient Bayesian-based multiview deconvolution. *Nat. Methods* **11**, 645-648 (2014).
26 Golden, T. *et al.* Dramatic age-related changes in nuclear and genome copy number in the nematode Caenorhabditis elegans. *Aging Cell* **6**, 179-188 (2007).
27 Klapper, M. *et al.* Fluorescence-based fixative and vital staining of lipid droplets in Caenorhabditis elegans reveal fat stores using microscopy and flow cytometry approaches. *J. Lipid Res.* **52**, 1281-1293 (2011).
28 Riesen, M. *et al.* MDL-1, a growth- and tumor-suppressor, slows aging and prevents germline hyperplasia and hypertrophy in C. elegans. *Aging* **6**, 98-117 (2014).
29 Kamath, R. S., Martinez-Campos, M., Zipperlen, P., Fraser, A. G. & Ahringer, J. Effectiveness of specific RNA-mediated interference through ingested double-stranded RNA in Caenorhabditis elegans. *Genome Biol.* **2**, RESEARCH0002 (2001).
30 Williams, G. C. Pleiotropy, natural selection and the evolution of senescence. *Evolution* **11**, 398-411 (1957).
31 Nesse, R. M. & Williams, G. C. *Why We Get Sick: The New Science of Darwinian Medicine*. (Random House, 1994).
32 Partridge, L. & Harvey, P. H. Methuselah among nematodes. *Nature* **366**, 404-405 (1993).
33 Gonczy, P. *et al.* Functional genomic analysis of cell division in C-elegans using RNAi of genes on chromosome III. *Nature* **408**, 331-336 (2000).
34 Korzelius, J. *et al.* C. elegans MCM-4 is a general DNA replication and checkpoint component with an epidermis-specific requirement for growth and viability. *Dev. Biol.* **350**, 358-369 (2011).
35 van den Heuvel, S. Cell-cycle regulation. *WormBook*, 1-16 (2005).

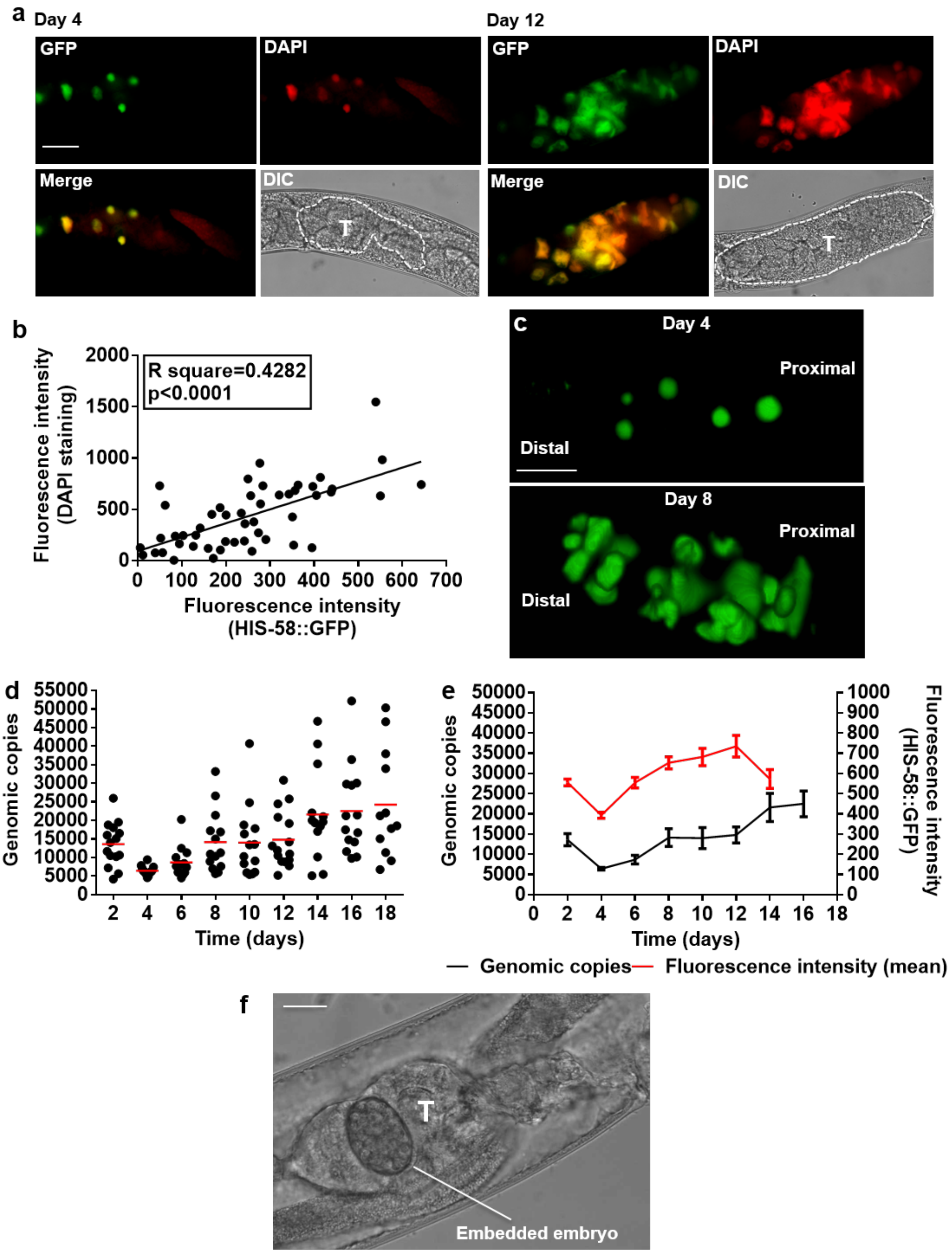

Figure S1 (**a**, **b**) Correspondence of HIS-58::GFP fluorescence and DNA content. (**a**) Comparison of HIS-58::GFP (chromatin marker) and DAPI fluorescence (i.e. DNA) in tumors. White dotted line delineates uterine tumor. Scale bar, 50 µm. (**b**) Correlation between HIS-58::GFP and DAPI fluorescence. Linear regression test, $p<0.0001$. (**c**) HIS-58::GFP-marked nuclei in tumor of day 4 and day 8 adults showing increased proximal nuclear hypertrophy (SPIM microscopy). Scale bar, 15 µm. (**d**) Change in genomic copy number with age. (**e**) Changes genomic copy number and nuclear HIS-58::GFP fluorescence with age (mean±s.e.m.). **a** - **e**: strain, GA1932 *unc-119(ed3); ltIs44 [pie-1p-mCherry::PH (PLC1deltal) + unc-119(+)]; ruIs32 [pie-1::GFP::H2B + unc-119(+)]*. (**f**) Embedded embryo within uterine tumor (day 8 adult). Scale bar, 25 µm.

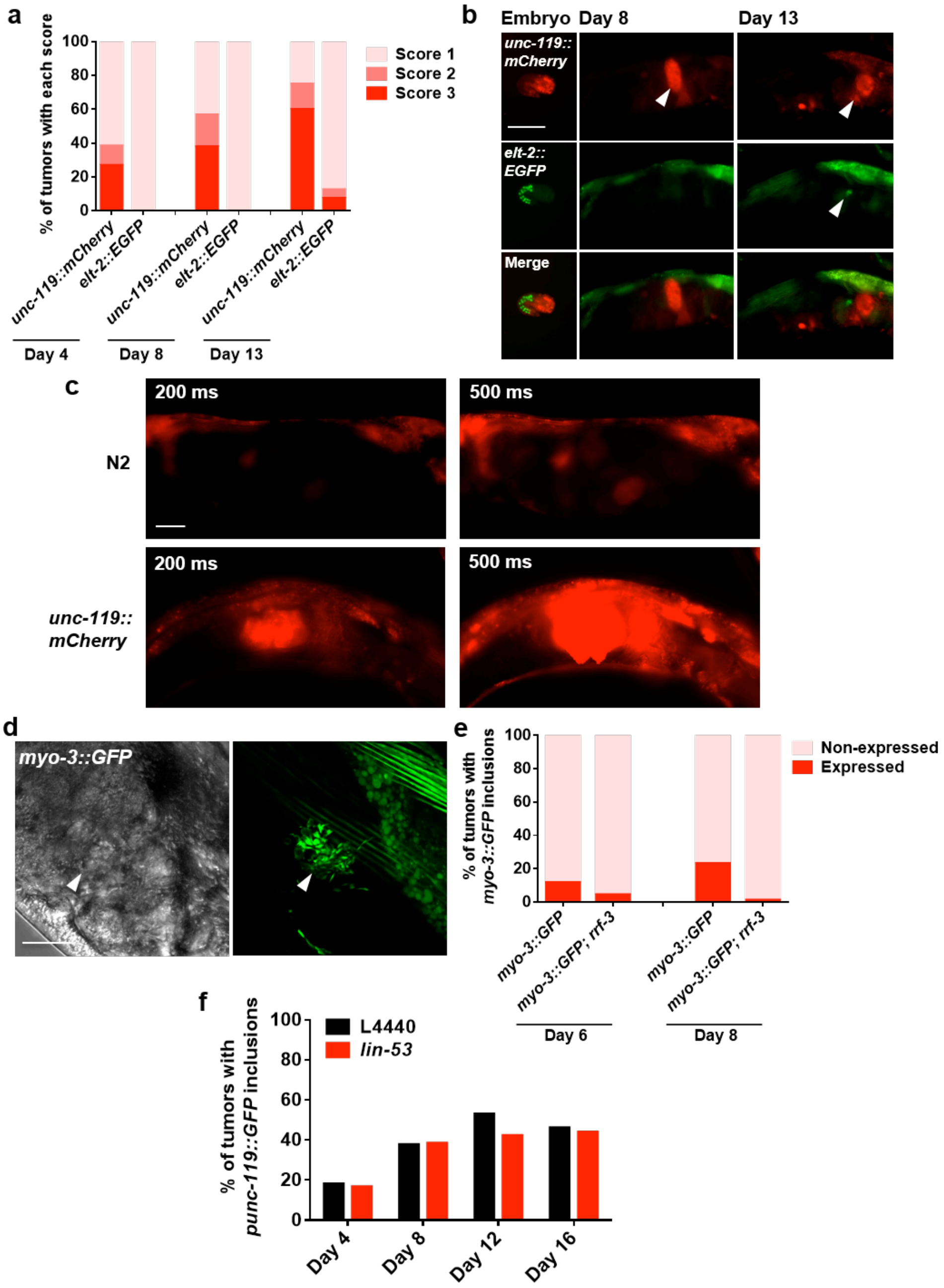

Figure S2 (**a**, **b**) Expression within the same tumor of *unc-119::mCherry* and *elt-2::EGFP*. (**a**) Frequency of tumors expressing fluorescent markers and different levels. One tumor per animal was scored. n=25-40. (**b**) Expression of *unc-119::GFP* and *elt-2::EGFP* in late embryos, and earlier and later stage tumors (representative images). Note that the day 13 tumor expresses both markers but at different sites. Scale bar, 50 μm. (**c**) Selected images of presence of red autofluorescence in N2, and mCherry fluorescence in *unc-119::mCherry* (MS1180) using different exposure times. Red autofluorescence in tumors is only visible if longer exposure times are used, therefore mCherry expression may readily be distinguished from autofluorescence. Scale bar, 25 μm. (**d**) Tumor of day 8 adult with *myo-3::GFP* inclusion (confocal microscopy). Scale bar, 25 μm. (**e**) Comparison of frequency of MYO-3::GFP positive inclusions in uterine tumor in *rrf-3(+)* and *rrf-3(b26)* strains. Rare, residual MYO-3::GFP positive inclusions in the latter resemble embedded embryos, suggesting possible incomplete penetrance of *rrf-3(b26)*. However, no larvae or dead eggs were seen on the NGM plates. N=3, n=50-66. (**f**) No effect of *lin-53* RNAi initiated at L4 on frequency of tumors expressing *punc-119::GFP*. N=2, n=18-36.

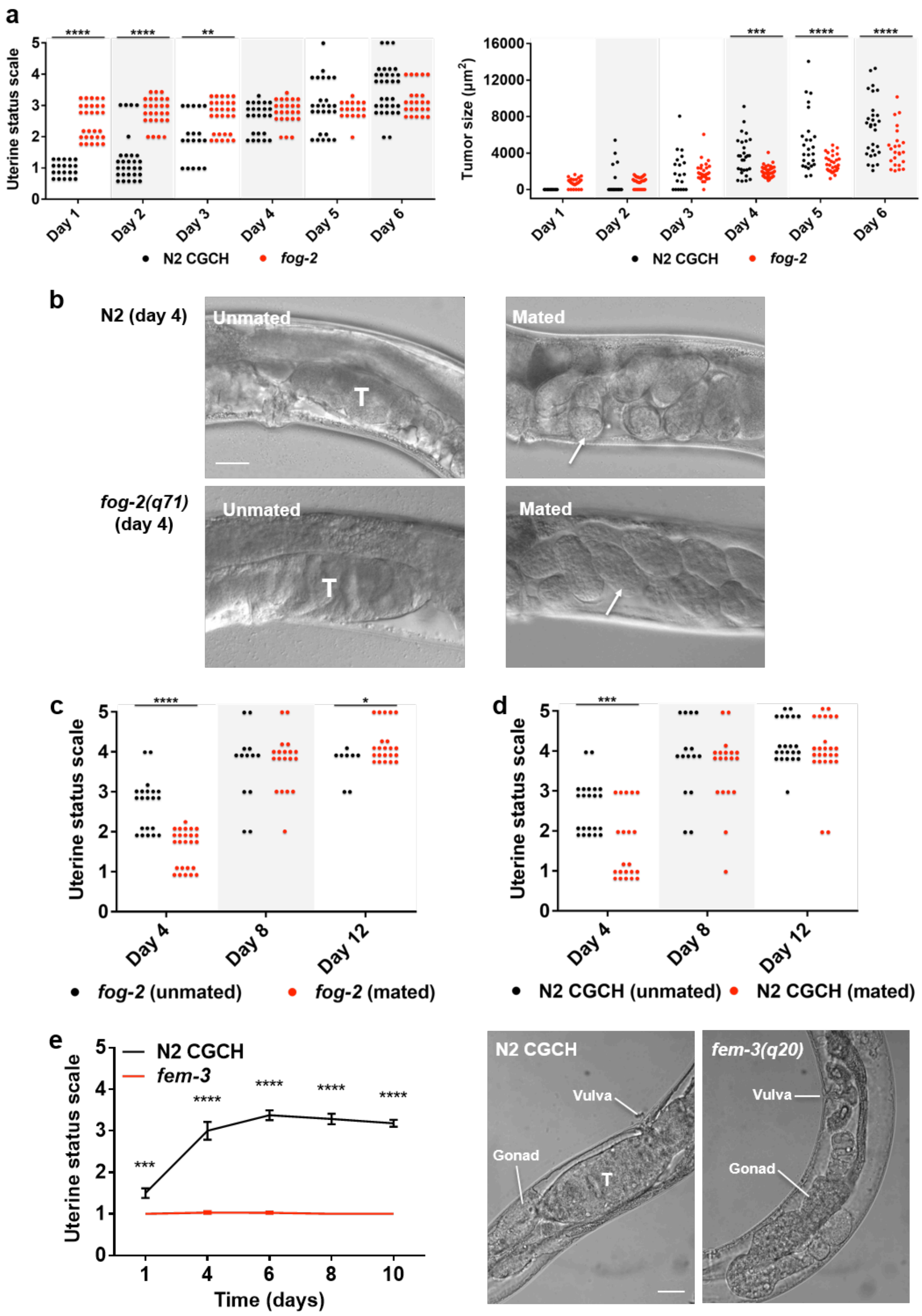

Figure S3 (**a**) Comparison of uterine status (left) and tumor size (right) in N2 and *fog-2(q71)* worms. Wilcoxon-Mann Whitney test, Sidak multiple comparison test, **, p<0.01; ***, p<0.001; ****, p<0.0001. *fog-2* causes earlier tumor development, but also reduced final tumor size suggesting a possible effect of prior sperm presence on later tumor growth. (**b-d**) Effects of *fog-2(q71)* and mating on timing of tumor development. (**b**) Selected images comparing N2 and *fog-2* tumors on day 4, and unmated and mated. Arrows, embryos; T, tumor. Scale bar, 25 µm. (**c**, **d**) Quantitation of tumor development unmated and mated animals. (**c**) *fog-2* females. (**d**) N2 hermaphrodites; the presence of tumors in some worms in the mated group on day 4 suggests that not all animals received male sperm. Evidence of sperm depletion by day 8 is consistent with the brief mating regimen used. Wilcoxon-Mann Whitney test, *, p<0.05; ***, p<0.001; ****, p<0.0001. (**e**) Tumors fail to grow in *fem-3(q20)* mutants. Quantitation of tumor development (left) (mean±s.e.m.); Wilcoxon-Mann Whitney test, ***, p<0.001; ****, p<0.0001. Selected images comparing N2 and *fem-3* tumors on day 6 (right). T, tumor. Scale bar, 25 µm.

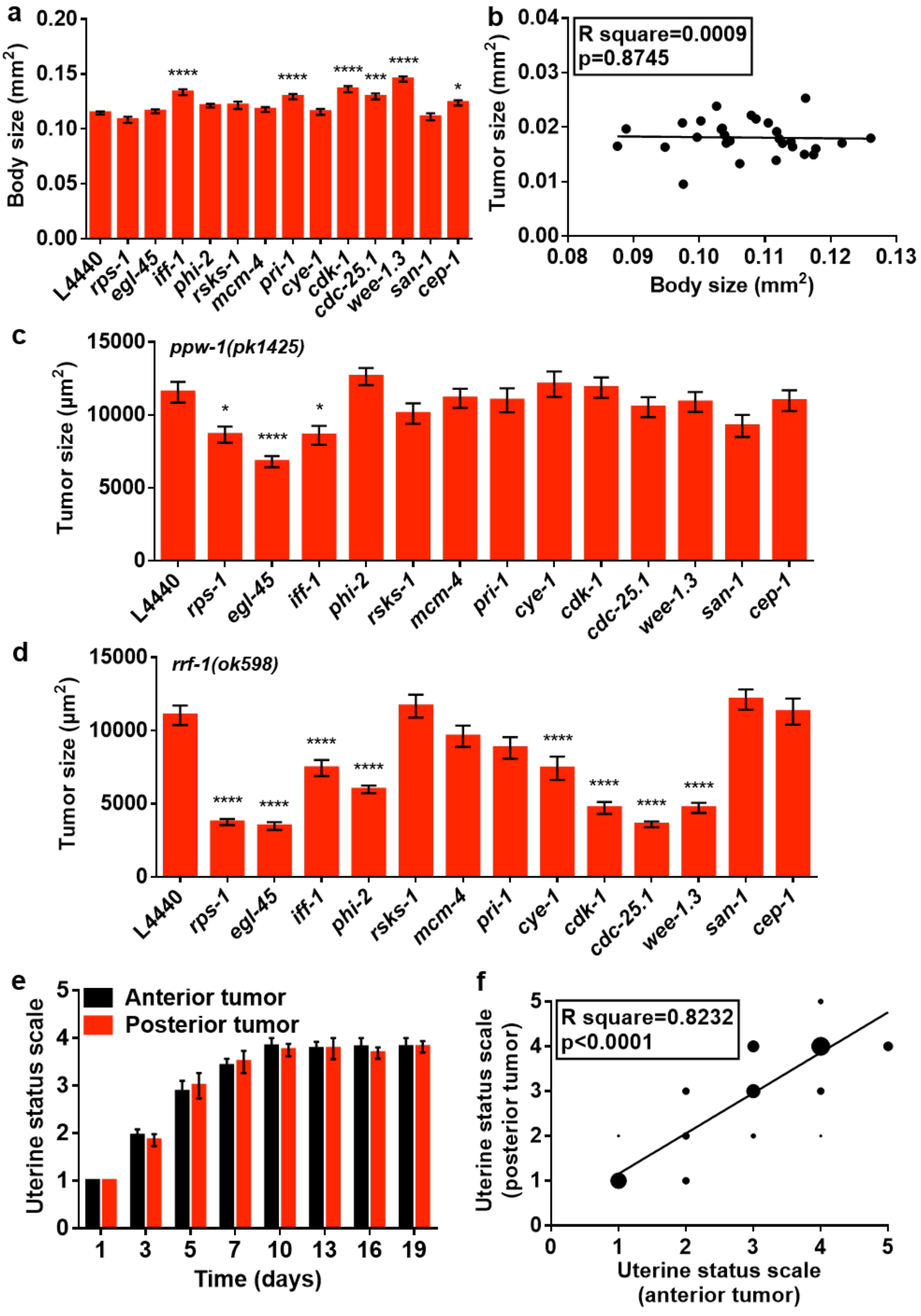

Figure S4 (**a**) RNAi did not decrease worm size (day 8). Data are mean±s.e.m.. (**b**) No correlation between body size (cross sectional area) and tumor size in N2 hermaphrodites (day 8). (**c**) Effects of somatic RNAi initiated at L4 on tumor size (mean±s.e.m.), measured on day 8 of adulthood. Dunnett multiple comparison test, *, p<0.05, ****, p<0.0001. All trials, n≥25. (**d**) Effects of germline RNAi initiated at L4 on tumor size (mean±s.e.m.), measured on day 8 of adulthood. ****, p<0.0001. All trials, n≥16. (**e**) Anterior uterine tumors are not larger or smaller overall than posterior uterine tumors (mean±s.e.m.). (**f**) Correlation between size of anterior and posterior tumors. Linear regression test, p<0.0001.

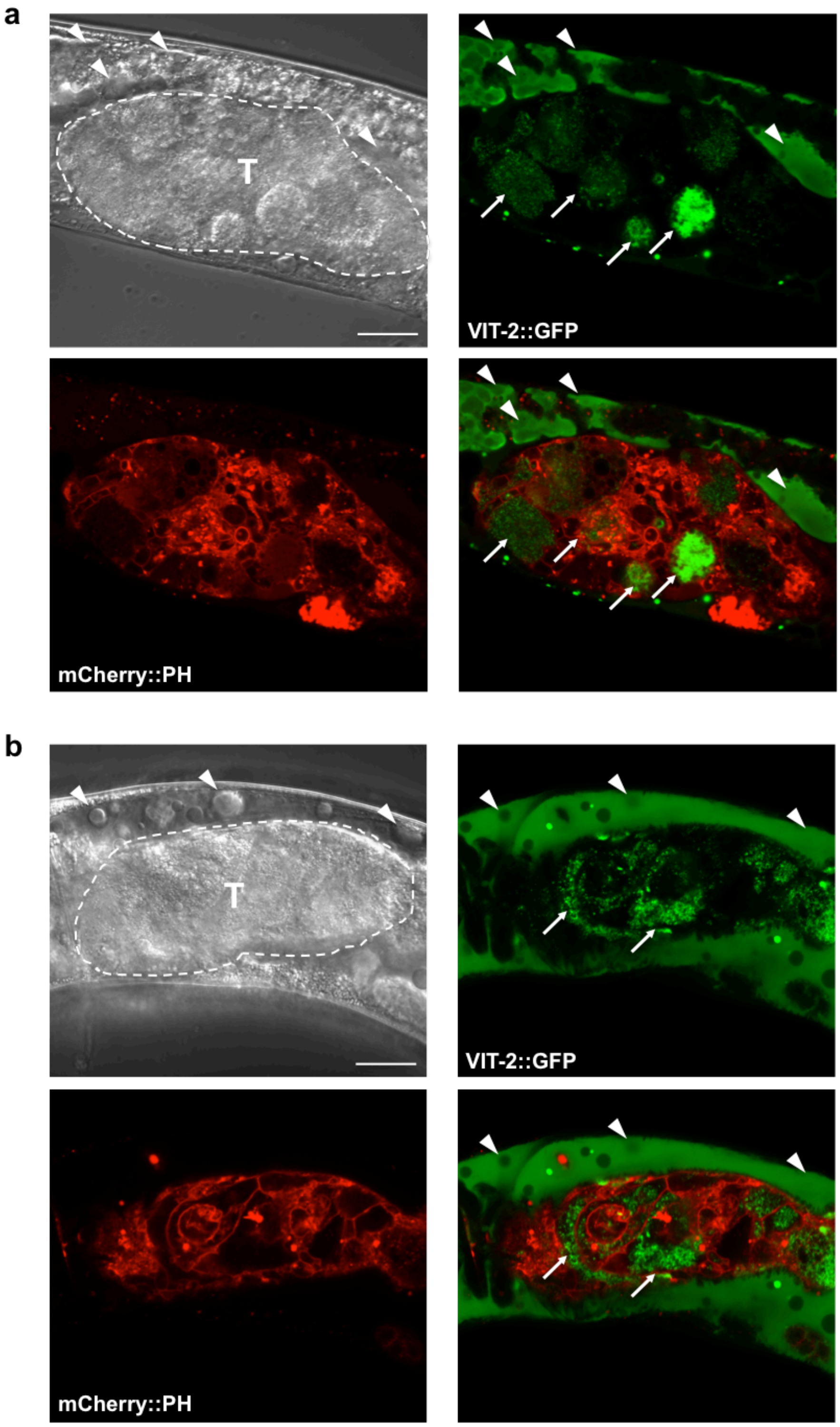

Figure S5 VIT-2::GFP accumulation in uterine tumors. (**a**, **b**) Two examples of uterine tumors labelled with both VIT::GFP and mCherry::PH (day 18 adults, confocal microscopy). VIT-2::GFP accumulation in oocytes within tumors (arrows) and in pseudocoelomic yolky pools (arrowheads). T, uterine tumor. Scale bar, 50 µm. See Video S3 for 3D image of (b).

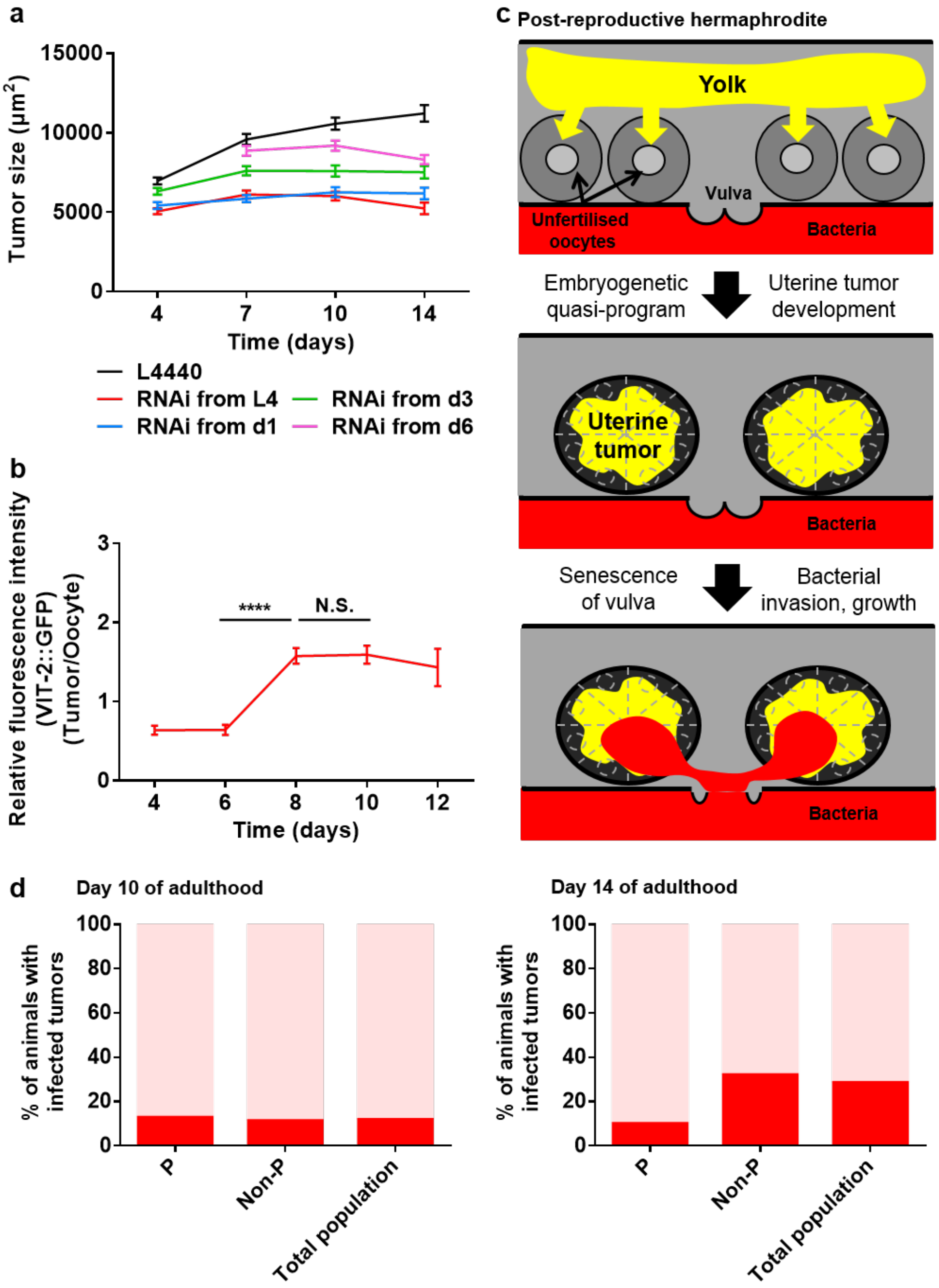

Figure S6 Relationship between other pathologies and uterine tumors. (**a-b**) Evidence that tumor growth is fed by accumulating yolk. (**a**) Blocking later yolk synthesis reduces tumor growth (*vit-5,-6* RNAi) (mean±s.e.m.). Summed data from 3 trials, all trials, n≥28. Statistically significant effects are seen in all cases (Table S3). (**b**) Yolk levels increase in growing tumors (25˚C) (mean±s.e.m.). VIT-2::GFP levels normalized to fluorescence in terminal oocyte distal to uterus. Tukey multiple comparison test, ****, p<0.0001, n=8-25. (**c, d**) *E. coli* infection of uterine tumors. (**c**) Hypothetical model for pathophysiology of infected tumor development. Unfertilised oocytes accumulate in the uterus, where growth into tumors is promoted by embryogenetic quasi-programs, and uptake of yolk from the body cavity yolk pools. In later life, aging of the vulva allows entry of *E. coli* (shown in red), which thrive in the nutrient-rich, yolky interior of tumors. (**d**) Worms with pharyngeal infection do not show a higher frequency of infected uteri. P, pharynx infected with dsRed-tagged *E. coli*; non-P, not infected[2]. The N2 line here used was CGCH[15]. Pink, uninfected; red, infected.

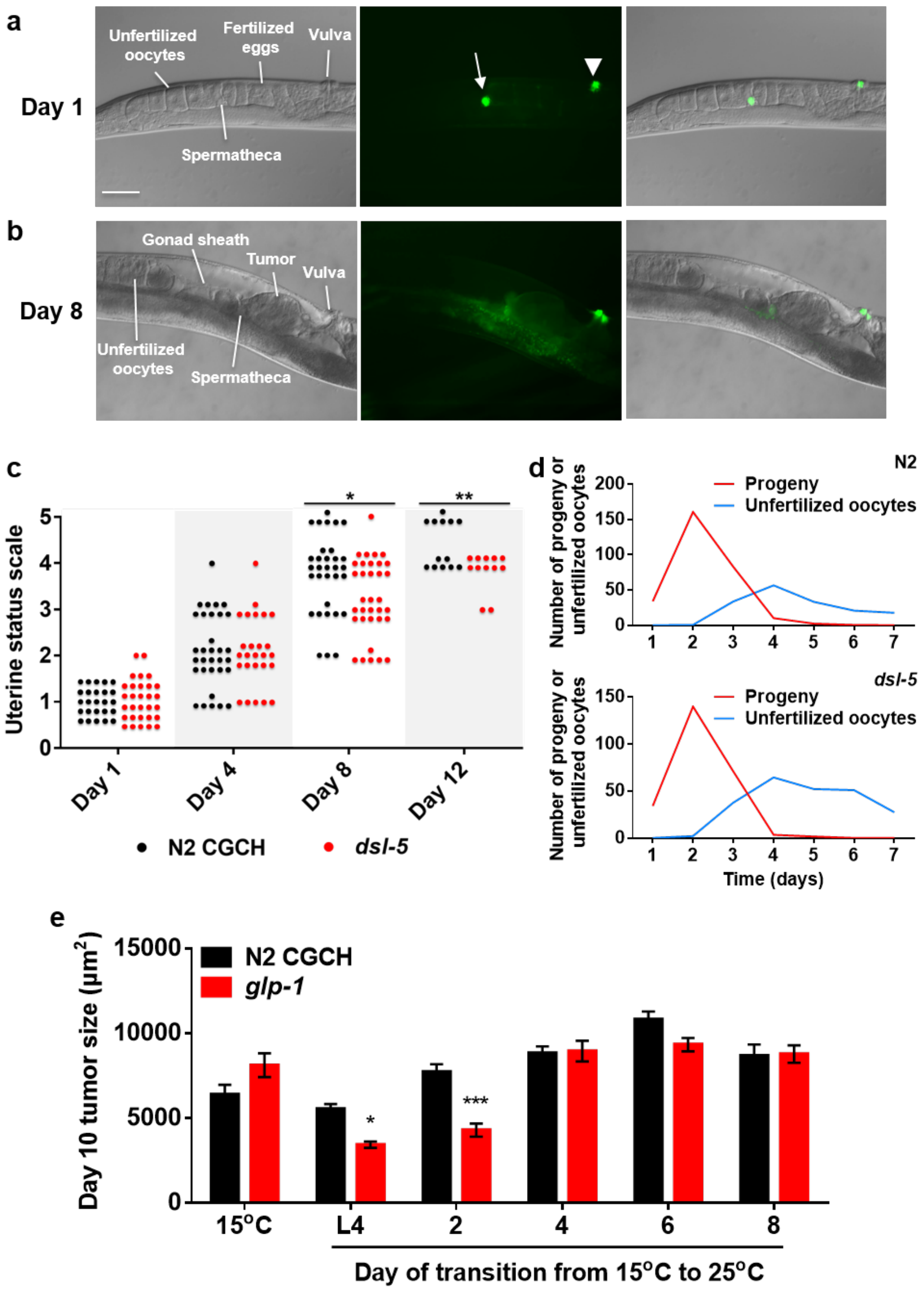

Figure S7 Is the uterus a latent tumor niche? (**a**, **b**) Tumors are contained within the uterus. (**a**) Day 1 of adulthood. Normal gonad with no tumor, and GFP expression at spermathecal uterine valve and vulva. (**b**) Day 8, GFP expression at spermathecal uterine valve indicates that the distal end of the tumor is within the uterus. Arrow, spermatheca; arrowhead, vulva. Scale bar, 50 μm. (**c**) *dsl-5(ok588)* reduces tumor size, in contrast to *apx-1(or3)* and *arg-1(ok3127)* (not shown). Wilcoxon-Mann Whitney test, *, $p<0.05$, **, $p<0.01$. (**d**) *dsl-5* does not delay sperm depletion; 11 broods examined per genotype. (**e**) No reduction in tumor size is detected when *glp-1(e2141ts)* is transferred to 25°C after sperm depletion (mean±s.e.m.), unpaired t-test, *, $p<0.05$, ***, $p<0.001$.

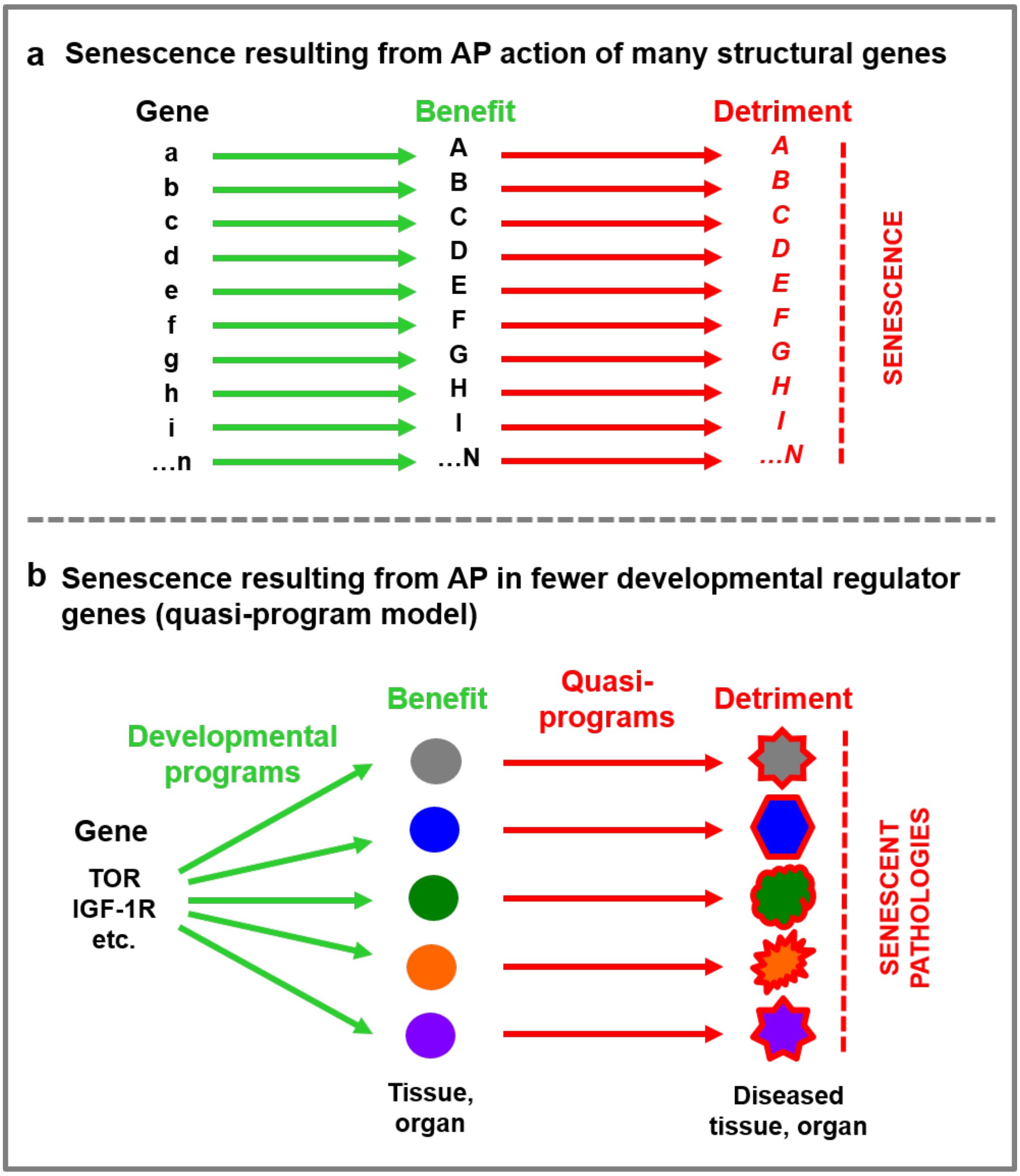

Figure S8: How antagonistic pleiotropy causes senescence: two models. (**a**) G.C. Williams viewed evolution as acting on countless individual structural genes (a-n) each with relatively narrow phenotypic impacts. To illustrate, he imagined a new allele of a gene involving in calcium deposition that enhances fitness by increasing bone development rate, but in later life promotes vascular calcification[30,31]. By this view, genes with global effects on aging are not expected[32]. But genes where mutation cause large increase in lifespan are often regulatory, particularly of growth and development. (**b**) M.V. Blagosklonny suggests that genes with major, global effects on aging control entire developmental and reproductive programs, and promote their futile run-on into non-adaptive quasi-programs, with pathogenic consequences. For example, as described in this study, complex developmental programs of oocyte maturation and embryogenesis, requiring action of numerous wild-type genes, run on to become quasi-programs causing uterine tumor formation. Mutation of the growth-promoting DAF-2 insulin/IGF-1 receptor inhibits uterine tumor development. Different colors of tissues, organs indicate distinct states of differentiation; e.g. in *C. elegans*, grey could be the germline, blue the intestine, and so on. TOR, target of rapamycin kinase; IGF-1R, insulin-like growth factor 1 receptor. Pale green and red indicate promotion of fitness and senescent pathology, respectively.

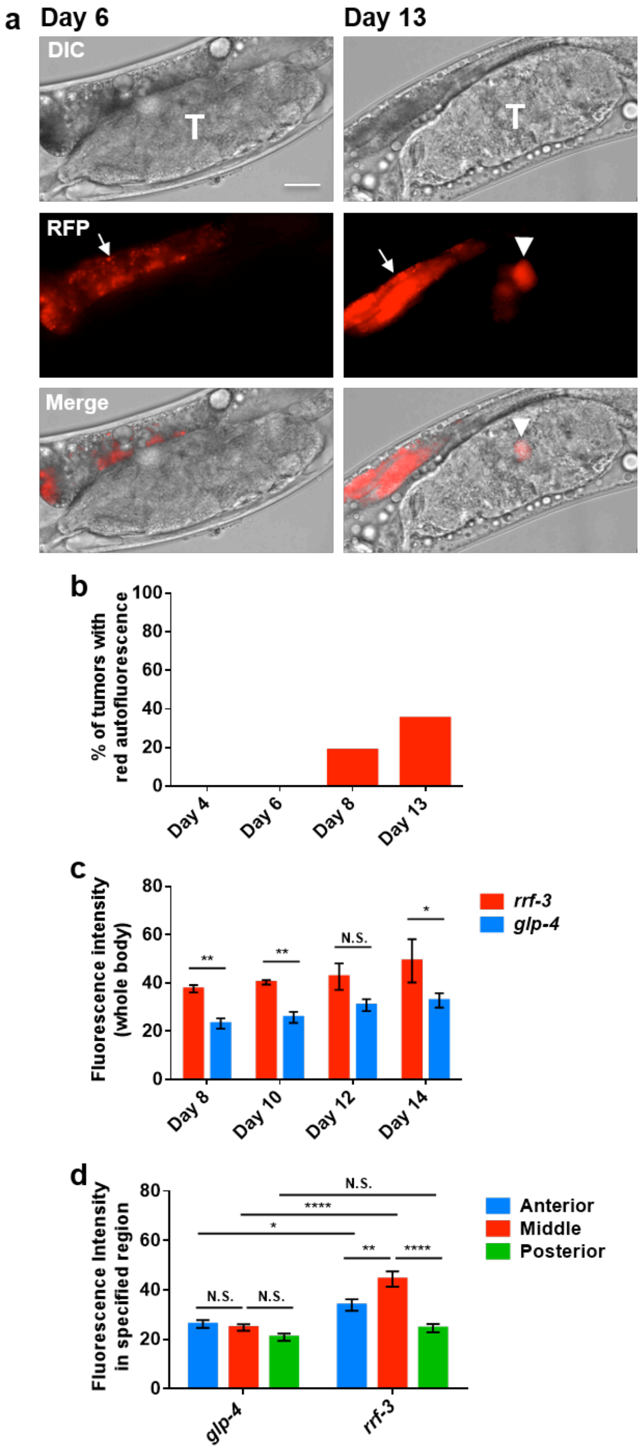

Figure S9 (**a**, **b**) Red fluorescence in older uterine tumors. (**a**) Red autofluorescence in tumor on day 13 (right) but not day 6 (left). T, tumor. Arrow, intestinal autofluorescence. Arrowhead, tumor autofluorescence. Strain, N2 CGCH. Scale bar, 25 µm. (**b**) Frequency of red fluorescence in uterine tumors with age. Day 4, n=48, day 6, n=60, day 8, n=59, day 13, n=57. (**c**, **d**) Greater red autofluorescence intensity in *rrf-3(b26)* (tumors) than *glp-4(bn2)* (no tumors). (**c**) Changes with age in whole worm red autofluorescence intensity. (**d**) Red autofluorescence intensity in anterior, mid- and posterior body regions. Worms were transferred from 15˚C to 25˚C at L4, and trials were performed at 25˚C. *glp-4* mutants, n=17; *rrf-3* mutants, n=18. **c**, **d**, Tukey multiple comparison test, Sidax multiple comparison test,*, p<0.05, **, p<0.01, ****, p<0.0001. Data are mean±s.e.m..

Table S1. Description of genes tested in RNAi experiments

**Gene name**	**Gene product identity, function**
rps-1	Ribosomal protein S3a, protein synthesis
egl-45	Translation initiation factor 3 subunit 10, protein synthesis
iff-1	Translation initiation factor eIF5A, protein synthesis
phi-2	Eukaryotic translation initiation factor eIF4A, protein synthesis
rsks-1	Ribosomal protein S6 kinase, protein synthesis
mcm-4	Minichromosome maintenance deficient 4 protein, DNA replication
pri-1	DNA polymerase alpha-primase subunit D, DNA replication
cye-1	G1/S cyclin, required for endoreduplication in intestinal cells, cell cycle
cdk-1	Cyclin-dependent kinase, romotes M phase entry/progression, cell cycle
cdc-25.1	CDK (cyclin-dependent kinase) activating dual specificity phosphatase, cell cycle
wee-1.3	CDK (cyclin-dependent kinase) inhibitory kinase, cell cycle
san-1	Mitotic checkpoint kinase, cell cycle
cep-1	*C. elegans* p53-like protein, DNA-damage checkpoint

Candidate tumor promoter genes, including genes specifying germline development and the cell cycle, were selected from those listed in several sources[33-35].

Table S2. Correlation between tumor size and nuclear morphology

		**Tumor size vs. nuclear morphology**	**Sample size**
Day 4	R square	0.2977	96
	P value	<0.0001	
Day 6	R square	0.1357	96
	P value	0.0002	
Day 8	R square	0.1938	96
	P value	<0.0001	
Day 10	R square	0.1059	76
	P value	0.0041	
Day 12	R square	0.1643	50
	P value	0.0035	
Day 14	R square	0.0132	32
	P value	0.5313	
Day 16	R square	0.1429	14
	P value	0.1827	

Summed data for three trials, all trials, n≥10. Linear regression analysis. Red font, $p < 0.05$.

Table S3. Statistical analysis of effects of *vit-5,-6* RNAi on tumor size

	Day 4	Day 7	Day 10	Day 14
L4440 (control)	--	--	--	--
RNAi from L4	p<0.0001	p<0.0001	p<0.0001	p<0.0001
RNAi from day 1	p=0.0003	p<0.0001	p<0.0001	p<0.0001
RNAi from day 3	p=0.28	p<0.0001	p<0.0001	p<0.0001
RNAi from day 6		p=0.20	p=0.0020	p<0.0001

Summed data for three trials, all trials, n≥28. Dunnett multiple comparison test. Red font, $p < 0.05$.
